# Emerging mechanistic insights of selective autophagy in hepatic diseases

**DOI:** 10.3389/fphar.2023.1149809

**Published:** 2023-03-16

**Authors:** Abdul Alim Al-Bari, Yuko Ito, Paul G. Thomes, Manoj B. Menon, Marina García-Macia, Raouf Fadel, Alfreda Stadlin, Nicholas Peake, MoezAlIslam Ezzat Faris, Nabil Eid, Daniel J. Klionsky

**Affiliations:** ^1^ Department of Pharmacy, Faculty of Science, University of Rajshahi, Rajshahi, Bangladesh; ^2^ Department of General and Gastroenterological Surgery, Osaka Medical and Pharmaceutical University, Osaka, Japan; ^3^ Department of Internal Medicine, University of Nebraska Medical Center, Omaha, NE, United States; ^4^ Kusuma School of Biological Sciences, Indian Institute of Technology Delhi, New Delhi, India; ^5^ Institute of Functional Biology and Genomics (IBFG), Universidad de Salamanca-CSIC, Institute of Biomedical Research of Salamanca (IBSAL), Hospital Universitario de Salamanca, Salamanca, Spain; ^6^ Department of Anatomy, College of Medicine and Medical Sciences, Arabian Gulf University, Al Manama, Bahrain; ^7^ Basic Medical Sciences Department, College of Medicine, Ajman university, Ajman, United Arab Emirates; ^8^ Biomolecular Sciences Research Centre, Sheffield Hallam University, Sheffield, United Kingdom; ^9^ Department of Clinical Nutrition and Dietetics, College of Health Sciences, University of Sharjah, United Arab Emirates; ^10^ Department of Anatomy, Division of Human Biology, School of Medicine, International Medical University, Kuala Lumpur, Malaysia; ^11^ Life Sciences Institute and Department of Molecular, Cellular and Developmental Biology, University of MI, Ann Arbor, MI, United States

**Keywords:** autophagy, lipophagy, liver disease, mitophagy, TFEB, virophagy

## Abstract

Macroautophagy (hereafter referred to as autophagy), a highly conserved metabolic process, regulates cellular homeostasis by degrading dysfunctional cytosolic constituents and invading pathogens *via* the lysosomal system. In addition, autophagy selectively recycles specific organelles such as damaged mitochondria (*via* mitophagy), and lipid droplets (LDs; *via* lipophagy) or eliminates specialized intracellular pathogenic microorganisms such as hepatitis B virus (HBV) and coronaviruses (*via* virophagy). Selective autophagy, particularly mitophagy, plays a key role in the preservation of healthy liver physiology, and its dysfunction is connected to the pathogenesis of a wide variety of liver diseases. For example, lipophagy has emerged as a defensive mechanism against chronic liver diseases. There is a prominent role for mitophagy and lipophagy in hepatic pathologies including non-alcoholic fatty liver disease (NAFLD), hepatocellular carcinoma (HCC), and drug-induced liver injury. Moreover, these selective autophagy pathways including virophagy are being investigated in the context of viral hepatitis and, more recently, the coronavirus disease 2019 (COVID-19)-associated hepatic pathologies. The interplay between diverse types of selective autophagy and its impact on liver diseases is briefly addressed. Thus, modulating selective autophagy (e.g., mitophagy) would seem to be effective in improving liver diseases. Considering the prominence of selective autophagy in liver physiology, this review summarizes the current understanding of the molecular mechanisms and functions of selective autophagy (mainly mitophagy and lipophagy) in liver physiology and pathophysiology. This may help in finding therapeutic interventions targeting hepatic diseases *via* manipulation of selective autophagy.

## 1 Introduction: Autophagy machinery at a glance

Cells produce huge quantities of waste products, and disposal through a unified degradation process is necessary to preserve cellular homoeostasis. Besides the ubiquitin (UB)-proteasome system (UPS) which regulates the degradation of short-lived proteins (Yin et al., 2020; Niture et al., 2021), lysosomal-dependent systems such as macroautophagy degrade various long-lived unwanted cytosolic materials (including damaged and superfluous organelles) and exogenous invading pathogens (Ke, 2020; Shojaei et al., 2020; Yang and Klionsky, 2020). For conserving cellular homeostasis, autophagy also controls cell survival pathways (Yang and Klionsky, 2020). To date, mammalian autophagy can be separated into three major types based on the cellular constituents that are delivered into the lysosome: macroautophagy (hereinafter referred to as autophagy), chaperone-mediated autophagy (CMA) and microautophagy (Hazari et al., 2020; Kim et al., 2020). The microautophagy pathway is the least characterized and involves the sequestration of cytoplasmic cargos directly at the surface of the lysosomal membrane; protrusion and/or membrane invagination followed by scission releases the cargo into the lysosomal lumen for subsequent degradation (Gatica et al., 2018; Lei and Klionsky, 2020). In addition, the endosomal sorting complexes required for transport (ESCRT) machinery also acts in the processes of microautophagy (Schäfer et al., 2020; Vietri et al., 2020). The selective degradation of proteins by CMA also involves uptake directly at the lysosomal surface; however, two key differences are that the targets of CMA are individual proteins, and these substrates must be unfolded and translocated directly across the lysosome membrane (Kaushik and Cuervo, 2018). CMA involves the recognition of proteins containing a KFERQ motif that binds to a molecular chaperone, HSPA8/HSC70 [heat shock protein family A (Hsp70) member 8]; these proteins are unfolded and then translocated into the lysosome through LAMP2A (lysosomal associated membrane protein 2A) in a process that involves lumenal HSPA8 along with other proteins, allowing the cargo to be degraded (Dash et al., 2019; Dong et al., 2021). Macroautophagy is the most well-defined form of autophagy in mammalian cells. Canonical autophagy involves the expression of the ATG (autophagy related) proteins, BECN1 (beclin 1) and MAP1LC3/LC3 (microtubule associated protein I light chain 3) and the formation of a sequestering compartment, the phagophore, that matures into a double-membrane autophagosome (Ueno and Komatsu, 2017; Levine and Kroemer, 2019). Non-canonical autophagy involves a subset of the core ATG machinery (Bello-Perez et al., 2020; Bhardwaj et al., 2020). For example, BECN1-independent autophagy can be stimulated by resveratrol (Scarlatti et al., 2008).

## 2 Regulation of the autophagy machinery

The term “autophagy” was coined by Christine de Duve (De Duve and Wattiaux, 1966; Klionsky, 2008) based on the observation of double-membranous dense bodies (detected in hepatocytes by TEM) as part of a cell-autonomous destruction process (Fenouille et al., 2017; Hansen et al., 2018; Viret et al., 2021). Shortly thereafter the concept of a vesicular process dependent on membranes that originated from those of intracellular organelles including the endoplasmic reticulum (ER) was developed (Gómez-Sánchez et al., 2021). The initiation of canonical autophagy is principally controlled by two classical autophagy master regulators (Al-Bari, 2020; Al-Bari and Xu, 2020). MTOR (mechanistic target of rapamycin kinase) complex 1 (MTORC1) inhibits two key complexes that are needed for autophagy induction: 1) The ULK (unc-51 like autophagy activating kinase) complex which is comprised of ULK1 or ULK2, RB1CC1/FIP200 (RB1 inducible coiled-coil 1), ATG13 and ATG101; and 2) the class III phosphatidylinositol 3-kinase (PtdIns3K) complex which is comprised of the lipid kinase PIK3C3/VPS34, PIK3R4/VPS15, BECN1, NRBF2 and, depending on the specific complex, ATG14, AMBRA1 (autophagy and beclin one regulator 1) or UVRAG (UV radiation resistance associated) (Condon and Sabatini, 2019). In mammalian cells, nutrient starvation typically inhibits the action of MTORC1 (Saxton and Sabatini, 2017; Condon and Sabatini, 2019), whereas in nutrient-rich situations, MTORC1 suppresses autophagy *via* phosphorylation of ULK1. The second major regulator of autophagy is AMP-activated protein kinase (AMPK), which activates ULK1 through stimulatory phosphorylation. MTOR and AMPK work along with various other factors as part of a complex network to attain precise levels of autophagic activity (Kim et al., 2008).

Inhibition of MTORC1 causes translocation of the ULK complex from the cytoplasm to the ER membrane (Jewell et al., 2013). Coordinately, the translocated ULK complex phosphorylates the class III PtdIns3K complex (Itakura and Mizushima, 2010; Matsunaga et al., 2010), resulting in the production of phosphatidylinositol-3-phosphate (PtdIns3P). PtdIns3P allows the recruitment of ZFYVE1/DFCP1 (zinc finger FYVE-type containing 1) and WIPI (WD repeat domain, phosphoinositide interacting)-family proteins to trigger phagophore formation. Furthermore, ATG9-mediated vesicle trafficking from the trans-Golgi network (TGN) to the ER and interaction with ATG2 supply the lipid constituents for phagophore nucleation and expansion (Mari et al., 2010; Yamamoto et al., 2012; Li et al., 2021; Xie et al., 2021). The expansion and maturation process involve two UB-like (UBL) conjugation cascade systems, which include ATG12–ATG5-ATG16L1 and the Atg8-family proteins (Ke, 2020). Finally, fusion of an autophagosome–or the product of an autophagosome first fusing with an endosome, termed an amphisome–with a lysosome requires SNARE and RAB proteins (Al-Bari and Xu, 2020). In the resulting autolysosome, hydrolytic enzymes degrade the autophagic cargo and release the end-products into the cytosol for the recycling of nutrients and energy production. Supplementary Figure S1 shows the various mechanisms of autophagy.

## 3 Selective autophagy

During the last decade, selective autophagy has been characterized as being distinct from non-selective (bulk) autophagy (Ke, 2020; Li et al., 2021; Xie et al., 2021). Selective autophagy has many functions including the protection of mammalian cells from organelle damage by acting in the turnover of dysfunctional organelles, termed “organellophagy” (Okamoto, 2014; Anding and Baehrecke, 2017; Gatica et al., 2018). Depending on the degraded substrates such as mitochondria, LDs, ER, peroxisomes, ribosomes, lysosomes, nuclei, invading pathogens (bacteria and viruses) as well as ferritin, selective autophagy has been divided further into mitophagy, lipophagy, reticulophagy, pexophagy, ribophagy, lysophagy, nucleophagy, xenophagy and ferritinophagy, respectively (Zhou et al., 2020; Faruk et al., 2021; Xu et al., 2021). Mitophagy, lipophagy and xenophagy are the best described and widely investigated types of selective autophagy.

### 3.1 Mitophagy machinery

As highly dynamic organelles, mitochondria undergo cycles of fusion and fission to control their remodeling and recycling of their constituents to support their mass and integrity (Eisner et al., 2018; Ke, 2020; Fenton et al., 2021). Mitochondria consist of two-layer membranes known as the inner mitochondrial membrane (IMM) and outer mitochondrial membrane (OMM), and the intermembrane space and the matrix, which mutually control biosynthesis, bioenergetics and cell signaling pathways. Healthy mitochondria are intracellular power factories that not only produce energy (ATP) *via* oxidative phosphorylation but also participate in other cellular functions (Palikaras et al., 2018; Spinelli and Haigis, 2018; Onishi et al., 2021). Conversely, defective mitochondria can produce excessive reactive oxygen species (ROS), which can damage cellular components including DNA. To maintain proper mitochondrial homeostasis, mitophagy must be precisely controlled and balanced with the biogenesis of new mitochondria (Zhu et al., 2013; Ding et al., 2021). The accumulation of defective mitochondria due to inadequate mitophagy may be part of the etiology for several diseases including cancer (Ding et al., 2021; Praharaj et al., 2021) due to impacts on several signaling pathways including inflammasome activation (Zhong et al., 2016). On the one hand, various stimulants, such as nutrient scarcity, hypoxia and viral infection induce mitophagy (Zhang et al., 2018; Ke, 2020; Malpartida et al., 2021). On the other hand, deregulated mitophagy can block the regeneration of healthy mitochondria causing the accumulation of defective mitochondria, which is associated with several pathological conditions (Malpartida et al., 2021) including inflammation (He et al., 2021; Onishi et al., 2021), cancer (Panigrahi et al., 2020; Rodrigues et al., 2020), liver injury (Aman et al., 2020; Ramachandran et al., 2021) and metabolic disorders (Su et al., 2021).

### 3.2 Mitophagy signaling pathways

**FIGURE 1:**
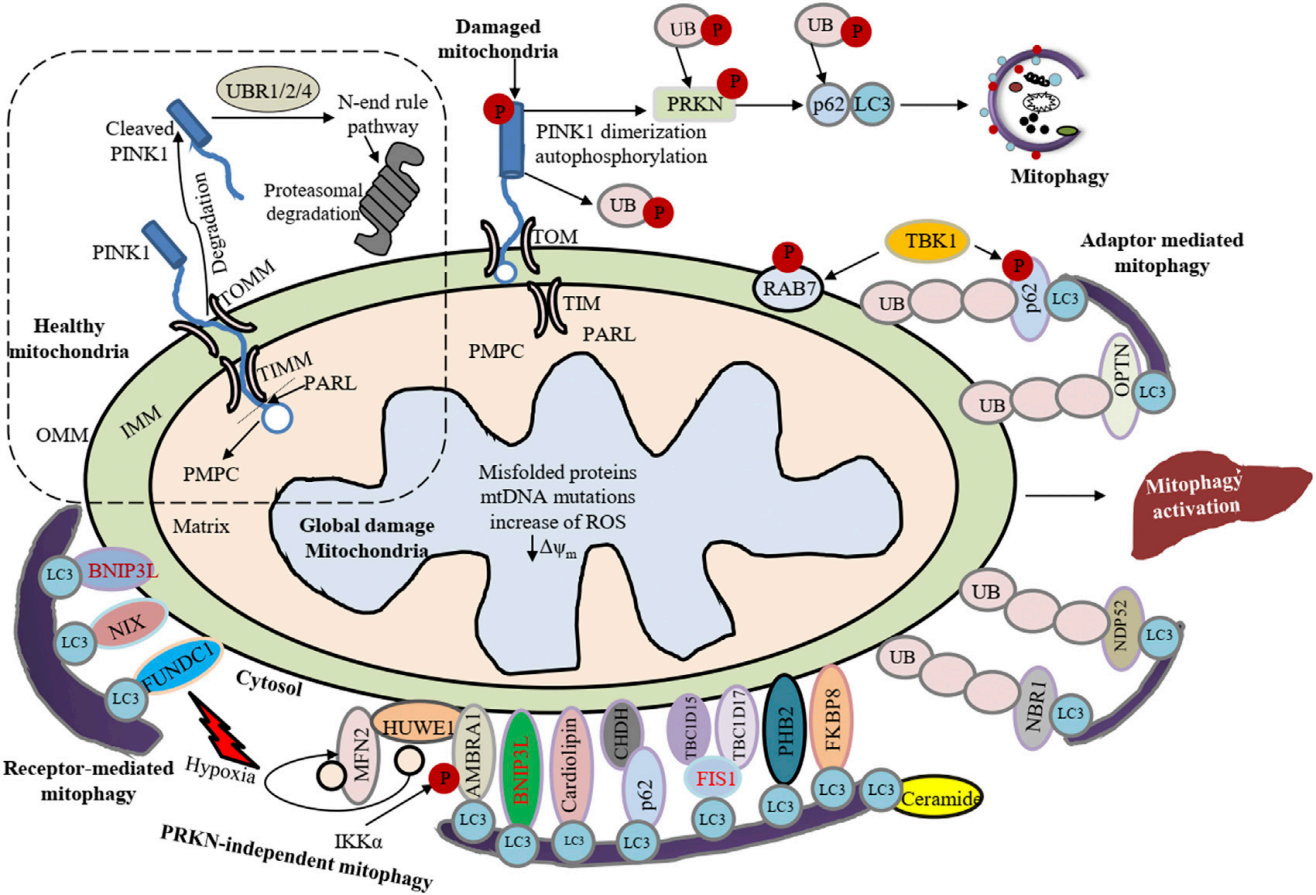
Summary of canonical mammalian mitophagy pathways. The cartoon represents classical mitophagy pathways: PINK1-PRKN/Parkin-dependent and receptor/adapter-facilitated mitophagy. In healthy mitochondria, the N terminus of PINK1 can be introduced into the IMM *via* TOMM and TIMM translocase complexes. The N terminus of the mitochondrial targeting sequence (MTS) and transmembrane (TM) segment are cleaved by PMPC/MPP and PARL, respectively. Subsequently, the cleaved PINK1 is exposed to the cytosol where the N-end-rule specific E3 enzymes UBR1, UBR2 and UBR4 recognize the destabilizing N-terminal phenylalanine residue of cleaved PINK1 for proteasomal degradation. Conversely, upon loss of mitochondrial membrane potential newly synthesized PINK1 is accumulated on the OMM, which can induce PRKN recruitment from the cytosol to mitochondria. Under hypoxia, FUNDC1, BNIP3 and BNIP3L recruit phagophores by directly interacting with LC3 through LIR domains. Upon induction of mitophagy, AMBRA1 mediates cytosolic HUWE1 translocation to mitochondria, leading to MFN2 degradation. Additionally, CHUK/IKKα kinase phosphorylates AMBRA1 (at S1014) and enables the binding between AMBRA1 and LC3 during mitophagy. PHB2, CL and BCL2L13 interact with LC3 and act as mitophagy receptors. Ceramide can act as a specific receptor for mitophagy by directly interacting with LC3. FKBP8 also interacts with LC3. CALCOCO2/NDP52 and OPTN act as the bridge connecting the UPS and autophagy because they can interact with both ubiquitin and LC3. NBR1 is replaceable for PRKN-mediated mitophagy regardless of SQSTM1/p62. TBK1-mediated phosphorylation endorses the recruitment of OPTN, CALCOCO2, and SQSTM1 to depolarized mitochondria. Under low MMP (Δψm), CHDH gathers in the OMM and binds with SQSTM1 through its PB1 domain, leading to CHDH-SQSTM1-LC3 complex formation that mediates mitophagy. TBC1D15 forms a complex with TBC1D17 and migrates to the OMM by interacting with FIS1 and the TBC1D15-TBC1D17 complex interacts with LC3.

**FIGURE 2:**
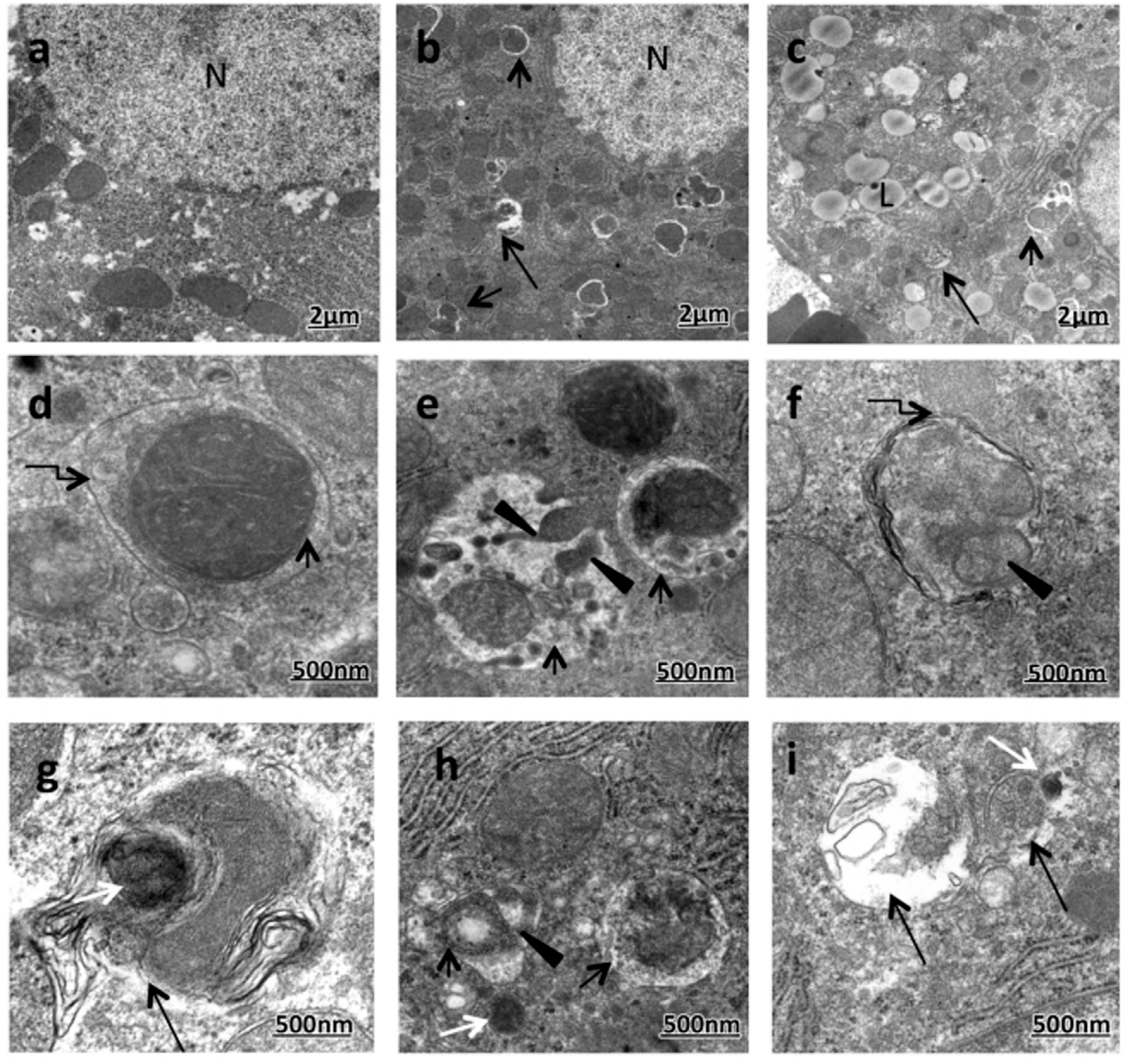
Enhanced formation of mitophagic vesicles (mitophagosomes and mitolysosomes) in the majority of ethanol-treated rats (ETRs) hepatocytes. **(A)**. Transmission electron microscopy showing control **(A)** and ETRs **(B–I)**. Short black arrows indicate mitophagosomes while long black arrows indicate mitolysosomes. Broken arrows show autophagic membranes. Black arrowheads indicate fragmented mitochondria while white arrows show lysosomes. N, nucleus; L, LD. (Eid et al., 2016; reprinted from Histology and Histopathology with permission).

Mitophagy participates in the elimination of damaged or excess mitochondria with the help of a bridge-like mitophagy receptor to degrade selective cargo (Gatica et al., 2018; Liu et al., 2020; Xie et al., 2021). These receptors generally have a conserved LC3-interacting region (LIR) composed of the core motif W/F/Y-X-X-L/V/I. Presently, two types of mitophagy receptors have been identified: soluble mitophagy receptors (SMRs) and membrane-attached mitophagy receptors (MMRs) (Figure 1). SMRs usually have single or double LIR motifs and one UB-interacting domain at the C terminus, while lacking a membrane translocation domain. The key SMRs include SQSTM1/p62, CALCOCO2/NDP52 (calcium binding and coiled-coil domain 2), OPTN (optineurin), NBR1 (NBR1 autophagy cargo receptor), and TAX1BP1 (Tax1 binding protein 1) (Lazarou et al., 2015; Li et al., 2021). The SMRs interact with cargoes through the UB-interacting domain and anchor themselves together with these cargoes to the phagophore membrane *via* LIR-mediated binding with the phosphatidylethanolamine-conjugated form of LC3 (LC3-II). In contrast, MMRs already reside on the mitochondria and do not directly bind with UB. MMRs such as BNIP3 (BCL2 interacting protein 3), BNIP3L/NIX (BCL2 interacting protein 3 like), BCL2L13 (BCL2 like 13), FUNDC1 (FUN14 domain containing 1) and PHB2 (prohibitin 2) directly bind to LC3 by their LIR motifs, thus recruiting mitochondria to the phagophore for initiation of mitophagy (Johansen and Lamark, 2011; Williams and Ding, 2018). The sequestering compartment of mitophagy is referred to as a mitophagosome; this compartment is essentially the same as an autophagosome, except that it forms in close apposition to the cargo and excludes bulk cytoplasm. The completed (closed) mitophagosome then shuttles to a lysosome for fusion (forming a mitolysosome) and cargo degradation (Figure 2). One of the best-characterized mechanisms of mammalian mitophagy involves the PINK1 (PTEN induced kinase 1) and PRKN/PARK2 (parkin RBR E3 ubiquitin protein ligase) pathway (Eid et al., 2016; Williams and Ding, 2018; Klionsky et al., 2021).

### 3.3 PINK1-PRKN-dependent mitophagy

Cytosolic PINK1-dependent stimulation of PRKN is one key pathway leading to mitophagy (Granatiero and Manfredi, 2019; Madruga et al., 2021). Under normal physiology, PINK1 translocates to the inner mitochondrial membrane through the OMM- and IMM-associated translocase complexes, TOMM and TIMM, respectively, where it is cleaved and inactivated by proteases such as PMPC/MPP (peptidase, mitochondrial processing) (Lazarou et al., 2012; Doblado et al., 2021) and PARL (presenilin associated rhomboid like) (Jin et al., 2010; Ke, 2020); the proteolyzed PINK1 is subsequently released into the cytosol where it is targeted by the N-end rule pathway machinery including UBR1 (ubiquitin protein ligase E3 component n-recognin 1), UBR2 and UBR4 for UPS degradation (Yamano and Youle, 2013; Pickrell and Youle, 2015; Doblado et al., 2021). Accordingly, the expression level of PINK1 is almost undetectably low on healthy mitochondria. However, under stresses such as mitochondrial damage, mutation of mitochondrial DNA (mtDNA) or increased ROS in mitochondria (Sekine, 2020), cleavage of PINK1 by PARL is terminated, and intact PINK1 resides on the OMM in interaction with the TOMM complex (Lazarou et al., 2012). The accumulated PINK1 kinase activity is induced by autophosphorylation, and in turn phosphorylates UB chains and PRKN at Ser65. This event is necessary to recruit PRKN from the cytosol to the OMM for inducing mitophagy (Pickrell and Youle, 2015; Eid et al., 2016; Matsuda, 2016; Doblado et al., 2021). Mitochondrial-resident PRKN enhances the ubiquitination of the OMM proteins that interact with SMRs to facilitate the recruitment of phagophores to damaged mitochondria. Based on this model, PINK1 acts as a sensor of mitochondrial damage, PRKN as a signal mediator and UB chains as the signal effectors (Harper et al., 2018; Doblado et al., 2021).

Interestingly, mitophagy is regulated by several cargo receptors. The most frequently considered PRKN substrates in the OMM are the mitochondrial fusion protein GTPases MFN1 (mitofusin 1) and MFN2, the mitochondrial trafficking proteins RHOT1/MIRO1 (ras homolog family member T1) and RHOT2/MIRO2, TOMM20 (translocase of outer mitochondrial membrane 20) and VDAC1 (voltage dependent anion channel 1) (Sarraf et al., 2013; Ma et al., 2020). PINK1-dependent phosphorylation positively regulates mitophagy, e.g., phosphorylation of MFN2 (at Thr111 and Ser442) and RHOT1 (at Thr298 and Thr299) by PINK1 induces mitophagy (Chen and Dorn, 2013; Safiulina et al., 2019; Ke, 2020). TBK1 (TANK binding kinase 1) also acts as a mitophagy inducer by phosphorylating mitophagy receptors. For example, TBK1 phosphorylates OPTN at Ser177 for stimulating its binding to LC3 proteins and at Ser473 and Ser513, endowing it with the capability to interact with the UB chain. Similarly, TBK1-facilitated autophosphorylation, SQSTM1/p62 phosphorylation at Ser403 and RAB7 phosphorylation at Ser72 have been found to control mitophagy (Ke, 2020). Moreover, mitochondrial RAB GTPase activating protein TBC1D9 (TBC1 domain family member 9), a Ca^2+^-binding protein, may stimulate mitophagy by inducing TBK1 (Ke, 2020). Once ubiquitinated, MFN1-MFN2 mediates proteasomal degradation resulting in an initial mitochondrial disintegration that assists in the segregation of damaged mitochondria from healthy ones as well as initiating mitophagy (Ma et al., 2020). PRKN also ubiquitinates RHOT1-RHOT2, which directly bind to PINK1 (Wang et al., 2011). In association with motor proteins that anchors mitochondria, RHOT1-RHOT2 proteins regulate Ca^2+^-dependent mitochondrial movement (Barazzuol et al., 2020). Here RHOT1-RHOT2 act as a Ca^2+^-dependent docking site and directly facilitate PRKN recruitment. Ca^2+^ binding causes the detachment of motor complexes from microtubules and leads to arrest the mitochondrial movement that initiates mitophagy (Wang and Schwarz, 2009; Safiulina et al., 2019; Doblado et al., 2021). In addition, PINK1-PRKN-facilitated ubiquitination of VDAC1 engages SMRs such as SQSTM1/p62 with damaged mitochondria. Then, SQSTM1/p62 is additionally recruited to LC^3+^ pre-mitophagosomes (phagophores) (Ma et al., 2020). PRKN also ubiquitinates BNIP3L/NIX, which permits BNIP3L to recruit NBR1, another SMR protein, to the damaged mitochondria to facilitate sequestration (Gao et al., 2015; Ma et al., 2020).

In addition, several IMM proteins in damaged mitochondria such as PHB2 bind to LC3 *via* the LIR domain to initiate mitophagy (Wei et al., 2017; Ma et al., 2020). PRKN also interacts with AMBRA1 (Cianfanelli et al., 2015; Li et al., 2021) at damaged mitochondria and AMBRA1 then additionally triggers the activity of the class III PtdIns3K complex for mitophagosome completion (Van Humbeeck et al., 2011; Li et al., 2021). It has been suggested that AMBRA1 controls the action of ULK1 by inducing the ubiquitination and self-assembly of ULK1 *via* TRAF6 (TNF receptor associated factor 6). Conversely, ULK1 also activates AMBRA1 by phosphorylation. These regulatory events constitute a positive feedback regulation loop to maintain mitophagy (Nazio et al., 2013). However, PINK1-PRKN-facilitated mitophagy can be inhibited by deubiquitinating enzymes, such as USP8 (ubiquitin specific peptidase 8), USP15, USP30, and USP35 (Wang L. et al., 2015; Xie et al., 2021). These enzymes eliminate the PRKN-facilitated ubiquitination signal from the damaged mitochondria. For example, TOMM20 is a known target of USP30 deubiquitinating activity and USP30 overexpression reverses PRKN ubiquitination of TOMM20, inhibiting mitophagy (Bingol et al., 2014; Cornelissen et al., 2014).

### 3.4 PRKN-independent mitophagy

PRKN is supposed to be a vital controller of mitophagy, yet accumulating data suggest that initiation of mitophagy may happen even with a deficiency of PRKN (Villa et al., 2018; Liu et al., 2019) or PINK1; mitophagy can be directly triggered by recruiting CALCOCO2 and/or OPTN to mitochondria (Lazarou et al., 2015). Several mitochondrial-resident mitophagy receptors such as BNIP3L, BNIP3 and FUNDC1 are stimulated under hypoxia, and CHDH (choline dehydrogenase) is induced upon disruption of mitochondrial membrane potential (MMP). These receptors further recruit phagophores through direct binding with LC3 independent of PRKN. In addition, a wide variety of UB E3 ligases such as MUL1 (mitochondrial E3 ubiquitin protein ligase 1) (Ke, 2020), ARIH1 (ariadne RBR E3 ubiquitin protein ligase 1), SMURF1 (SMAD specific E3 ubiquitin protein ligase 1), HUWE1 (HECT, UBA and WWE domain containing E3 ubiquitin protein ligase 1), AMFR/gp78 (autocrine motility factor receptor) and SIAH1 (siah E3 ubiquitin protein ligase 1) are involved in mitophagy progression (Ke, 2020). Thus, PRKN-independent mitophagy can be divided into receptor-facilitated and UB ligase-facilitated mitophagy.

### 3.5 Receptor-mediated mitophagy

BNIP3L/NIX localizes to the OMM and is identified as a member of the BH3-only protein family with pro-apoptotic activity. A small GTPase, RHEB, is recruited to the OMM with oxidative phosphorylation activity and promotes mitophagic activity through interaction with BNIP3L and LC3-II. Under hypoxia, BNIP3L binds to Atg8-family proteins such as LC3 through its LIR motif for inducing mitophagy during reticulocyte maturation (Novak et al., 2010; Ma et al., 2020; Li et al., 2021). Phosphorylation of BNIP3 at Ser17 and Ser24 flanking the LIR motif promotes its interaction with LC3 facilitating mitophagy. BCL2L13 belonging to the BCL2 family has a single transmembrane (TM) domain as an OMM protein and two WXXI motifs, permitting it to interact with LC3 and it induces mitophagy independent of PRKN (Murakawa et al., 2015). Under hypoxia, FUNDC1 directly binds *via* its LIR motif to LC3 and induces PRKN-independent mitophagy (Liu et al., 2012; Ma et al., 2020; Wang D. et al., 2020). BCL2L13 and FUNDC1 can also bind and localize ULK1 to the mitochondria directly (Wu et al., 2014; Murakawa et al., 2019; Killackey et al., 2020). PGAM5 (PGAM family member 5, mitochondrial serine/threonine protein phosphatase) in mitochondria de-phosphorylates FUNDC1 (at Ser13), and ULK1 and SRC kinase phosphorylate FUNDC1 (at Ser17 and Tyr18, respectively) which initiates hypoxia-induced FUNDC1-mediated mitophagy (Chen et al., 2014; Ma et al., 2020; Wang Y. et al., 2020). In addition, MARCH5 (membrane associated ring-CH-type finger 5) E3 ligase promotes FUNDC1 degradation by UB-mediated proteasomal modification, and ultimately prevents the occurrence of mitophagy (Chen et al., 2017; Villa et al., 2018). Moreover, TBC1D15 (TBC1 domain family member 15) forms a complex with TBC1D17 and translocates to the OMM by binding to FIS1 (fission, mitochondrial 1) (Losón et al., 2013). The TBC1D15-TBC1D17 complex then connects with LC3 and promotes mitophagy (Yamano et al., 2014; Doblado et al., 2021). Under normal conditions, CHDH is found in both the IMM and OMM. When MMP is disrupted, accumulated CHDH on the OMM binds to SQSTM1 *via* its Phox and Bem1 (PB1) domain, leading to CHDH-SQSTM1-LC3 complex formation and mitophagy induction (Park et al., 2014; Doblado et al., 2021).

In addition to the mitophagy protein receptors, several phospholipids can interact with LC3 and act as mitophagy receptors for regulation of mitochondrial dynamics including fission and fusion. For example, the IMM-oriented phospholipid, cardiolipin (CL) translocates to the OMM in the case of mitochondrial damage (Chu et al., 2013; Killackey et al., 2020). MFN (mitofusion) is activated by PLD6/mitoPLD (phospholipase D family member 6) that converts CL into phosphatidic acid. OMM-oriented CL directly interacts with LC3 to induce mitophagy (Chu et al., 2013). Furthermore, another sphingolipid ceramide directly interacts with LC3 to engage mitophagosomes (Sentelle et al., 2012). In cellular stress conditions, the IMM fusion protein full-length or long OPA1 (OPA1, mitochondrial dynamin like GTPase; L-OPA1) is cleaved to short (S)-OPA1, promoting OMM permeabilization and CYCS/cytochrome c release (Ni et al., 2015; Wang et al., 2018). Additionally, mitochondrial fission (mitofission) is thought to be important for mitophagy. For example, mitofission of the OMM is regulated by DNM1L/DRP1 (dynamin one like) and its four mitochondrial receptor proteins: FIS1, MFF (mitochondrial fission factor) and MIEF1/MID51 (mitochondrial elongation factor 1)-MIEF2/MID49 (Losón et al., 2013; Ni et al., 2015; Osellame et al., 2016; Doblado et al., 2021). The translocation of cytosolic DNM1L to mitochondria stimulates fission (Kageyama et al., 2014; Li et al., 2021) resulting in mitochondrial constriction leading to eventual division and fragmentation *via* induction of mitophagy. The OMM-oriented FKBP8 (FKBP prolyl isomerase 8) is another mitophagy receptor (Kageyama et al., 2014; Bhujabal et al., 2017). Mechanistically, FKBP8 acts as an LC3-interacting protein *via* its LIR motif to induce mitophagy *via* PRKN-independent mitophagy. However, mitochondrial FKBP8 also translocates to the ER upon PRKN-mediated mitophagy and thus escapes from degradation (Bhujabal et al., 2017); a direct involvement of FKBP8 in mitophagy, however, has not yet been identified. Another report suggests that activation of PINK1 is more closely related to DNM1L-mediated mitofission and quality control independent of PRKN that leads to metabolic diseases such as insulin resistance, type 2 diabetes, and fatty liver (Axelrod et al., 2021)

### 3.6 Ubiquitin ligase-mediated mitophagy

OMM-resident MUL1 can induce mitophagy in response to damaged mitochondria in mammalian cells. Knockdown of MUL1 in PRKN-expressing cells renders them incapable of mitochondrial PRKN translocation following depolarization (Yun et al., 2014; Xie et al., 2021), indicating that the action of MUL1 may be PRKN independent. ARIH1 induce mitophagy in a PRKN-independent manner in cancer cells (Villa et al., 2017) resulting in cancer cell resistance to anti-cancer therapy (Liu et al., 2019). SMURF1 may also regulate mitophagy (Choubey et al., 2014; Doblado et al., 2021). *Smurf1*-knockout mice have an augmented damaged mitochondrial accumulation in liver, and studies suggest that SMURF1 is necessary for mitophagosome formation (Ma et al., 2020). Further, the protein AMFR/GP78 is involved in ER-associated degradation. Upregulated AMFR expression enhances MFN1-MFN2 ubiquitination leading to proteasomal degradation as part of an initiation of mitophagy. In contrast, AMFR knockdown increases the expression of MFN1-MFN2 and decreases PRKN-independent MMP-induced mitophagy (Bingol et al., 2014). HUWE1 stimulates mitophagy by promoting CHUK/IKK-α (component of inhibitor of nuclear factor kappa B kinase complex)-mediated AMBRA1 phosphorylation (at Ser1014) and degradation of MFN2. Phosphorylation of AMBRA1 promotes its interaction with LC3 and subsequently enhances PINK1-PRKN-independent mitophagy (Di Rita et al., 2018; Strappazzon et al., 2020).

## 4 Lipophagy and its regulation

Fatty acids (FAs) are critical cellular components, as they organize basic constituents of biological membranes and can be utilized as energy substrates *via* β-oxidation within mitochondria. However, accumulation of FAs can be detrimental to mammalian cells due to their lipotoxicity; thus, cells transform these FAs into neutral lipids for storage in highly dynamic specialized organelles called LDs (Grefhorst et al., 2021). Hepatocytes act as a key cellular storehouse for neutral lipids in the form of intracellular triglycerides (TGs) and cholesterol esters enclosed in LDs (Czaja et al., 2013). Until 2009 it was generally thought that the metabolism of these stored lipids occurs solely by cytoplasmic neutral lipases and by LIPA/LAL (lipase A, lysosomal acid type), but do not undergo autophagy. However, Singh et al. (2009) verified the existence of a selective autophagic mechanism called lipophagy for specific breakdown of LD stores (Singh et al., 2009). Thus, regulation of intracellular LD metabolism in hepatocytes is mediated by both lipolysis and lipophagy but the different signaling pathways of these processes remain unclear (Eid et al., 2013a; Eid et al., 2013b; Niture et al., 2021).

**FIGURE 3:**
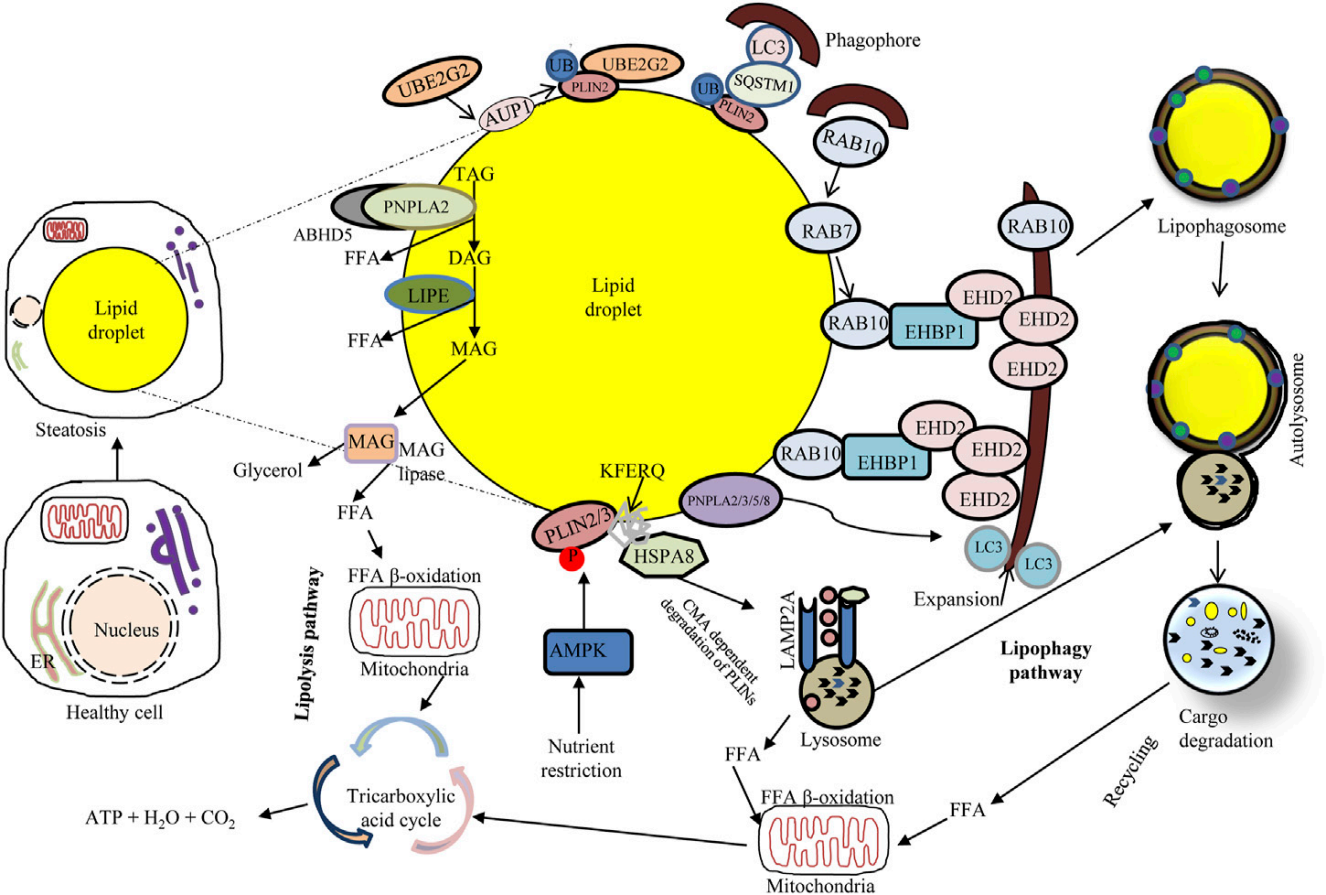
Overview of major lipid metabolism pathways: connecting lipophagy and lipolysis. Lipophagy involves small cytosolic LDs (cLDs) or sequestering portions of large cLDs. Lipophagosomes (autophagosomes containing LDs) deliver cLD cargo to lysosomes, wherein LIPA/LAL degrades the lipid cargo and subsequently releases FFAs that undergo mitochondrial β-oxidation to produce ATP. Activation of chaperone-mediated autophagy degrades the cLD coat proteins PLIN2 and PLIN3 through the coordinated action of HSPA8; the cLD surface becomes accessible to neutral lipolysis by PNPLA2/ATGL in complex with LD-binding protein ABHD5/CGI-58, which hydrolyzes the cLD triacylglycerols to generate FFAs. Nutrient deprivation induces AMPK that inhibits MTORC1 and triggers autophagy. Upon nutrient deprivation, the expression of RAB7 increases; in that event RAB7 directly facilitates lipophagy and also recruits RAB10 to the LD. RAB10 forms a complex with EHBP1 and EHD2 to initiate lipophagy, potentially through membrane expansion along the LD surface. The LD surface protein PLIN2 can bind SQSTM1, a selective autophagy receptor which binds LC3 on phagophores. AUP1 recruits the ubiquitin ligase UBE2G2 *via* its G2BR domain which ubiquitinates LD surface proteins. This ubiquitination may facilitate lipophagy through a to-be-determined selective autophagy receptor binding to phagophores.

The lipolytic pathway relies on the direct activation of cytosolic lipases including PNPLA2/ATGL (patatin like phospholipase domain containing 2), LIPE/HSL (lipase E, hormone sensitive type) and MGLL (monoglyceride lipase) working together with regulatory protein factors (e.g., an activator protein of PNPLA2/ATGL called ABHD5/CGI-58 [abhydrolase domain containing 5, lysophosphatidic acid acyltransferase]) (Zechner et al., 2012; Kloska et al., 2020; Niture et al., 2021) (Figure 3). Under normal fed conditions, LDs mostly store triacylglycerol (TAG) in adipose tissue, and lipolysis causes the hydrolysis of ester bonds between long-chain FAs and the glycerol backbone (Li et al., 2021). During the early stage of this process, PRKA/protein kinase A phosphorylates PLIN (perilipin) proteins leading to their proteasomal degradation. Different PLINs seem to be able to distinguish between diverse sizes and structures of LDs (Li et al., 2021). In CMA, the LD coat proteins PLIN2 and PLIN3 are degraded through the harmonized action of HSPA8/HSC70 and the membrane channel LAMP2A (Kaushik and Cuervo, 2016; Yang et al., 2019). The phosphorylation of PLINs releases ABHD5, which specifically induces PNPLA2/ATGL which in turn catalyzes the hydrolysis of TAG to form diacylglycerols (DAGs) and free FAs (FFAs) (Schreiber et al., 2019). The following stage of lipolysis is dependent on the stimulation of multi-purpose enzymes such as LIPE/HSL. Hydrolysis of DAGs by LIPE/HSL yields monoacylglycerol (MAG) and FFAs (Schott et al., 2019). The last stage of the lipolysis cascade is dependent on MGLL stimulation that cleaves MAGs, generating glycerol and FAs (Kloska et al., 2020). FFAs released through this process can become substrates for mitochondrial β-oxidation or act as effective signaling molecules for regulating several cellular processes (Schott et al., 2019).

**FIGURE 4:**
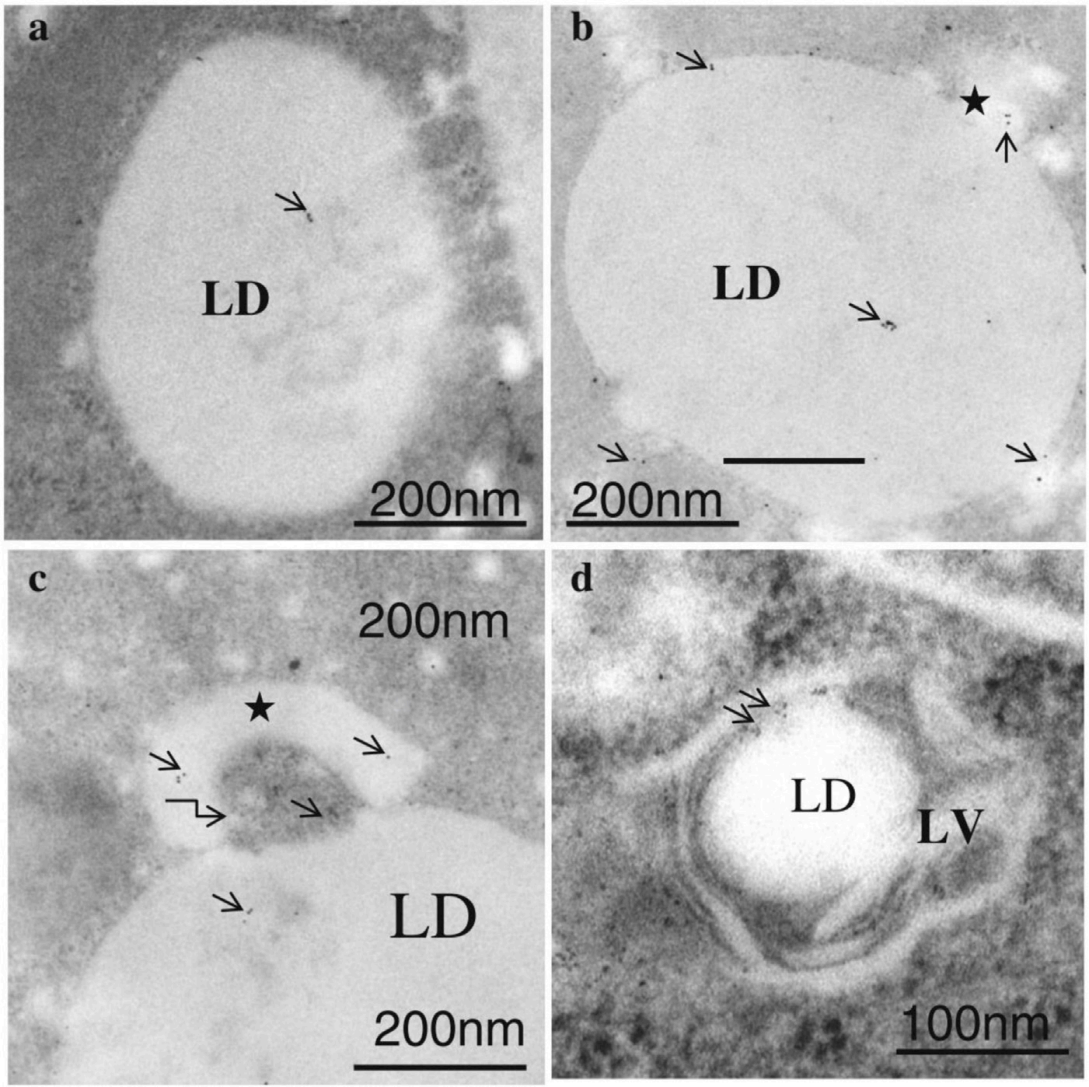
Immunogold labeling of LC3 in lipophagosomes of ETRs hepatocytes **(B–D)** and control **(A)**. Short arrows indicate LC3 immunogold particles. Stars show smaller LDs originating from a larger LD. The curved arrow in c marks a membranous structure. LD: lipid droplet; LV: lipophagic vacuole. (Eid et al., 2013a; reprinted from JMH with permission).

In lipophagy (Figure 3) the specific turnover of LDs occurs *via* the autophagy-lysosome system, through the function of acid lipases resident to the autolysosome. Lipophagy thus functions as an alternative to classical cytosolic lipase-mediated LD degradation (Li et al., 2021). To date, several cytoplasmic adipose TG lipases e.g., PNPLA2/ATGL, PNPLA3, PNPLA5, and PNPLA8 have been recognized as receptors of lipophagy (Li et al., 2021). Remarkably, both lipolysis and lipophagy can be controlled by PNPLA2/ATGL (Zechner et al., 2017). LC3 directly interacts with PNPLA2/ATGL and LIPE/HSL at the LD surface. Under conditions of nutrient deprivation, LC3 interacts with PNPLA2/ATGL through a LIR domain allowing translocation of the latter to the LD surface for assisting hydrolysis of TAG (Kloska et al., 2020). Moreover, PNPLA2/ATGL-activated lipophagy hastens LD breakdown and FFA oxidation *via* the promotion of SIRT1 (sirtuin 1) activity. Another lipase, PNPLA8, can also bind to LC3 to trigger lipophagy in a high-fat diet (HFD) mouse model. Furthermore, PNPLA3 is necessary for lipophagy in serum-starved hepatocytes and PNPLA5 participates in both lipophagy and mitophagy (Li et al., 2021). The ultrastructural features of lipophagy are shown in Figure 4.

Numerous members of the RAB GTPase family have been identified as important mediators of LD catabolism events. Upon nutrient deprivation, RAB7 is a key player associated with both LDs and autophagic membranes, which becomes active and stimulates the movement of lysosomes near LDs for their targeted degradation *via* lipophagy (Kloska et al., 2020). Another GTPase, RAB10, is confined on the LD surface and lipophagosomes during starvation mediated by RAB7, and potentially participates in lipophagy. RAB10 may act downstream of RAB7 as a constituent of the complex that stimulates the sequestration of LDs during lipophagy progression. Accordingly, RAB10 engages the adapter protein EHBP1 (EH domain binding protein 1) with the ATPase EHD2 (EH domain containing 2) that mechanically drives the expansion of the phagophore membrane around the LDs for engulfment (Kloska et al., 2020; Wu et al., 2021). Furthermore, polyubiquitination can modify proteins and function as a signal to induce lipophagy. For example, following LDs ubiquitination, interaction between LDs-associated ubiquitinated AUP1 (AUP1 lipid droplet regulating VLDL assembly factor) and UBE2G2 (ubiquitin conjugating enzyme E2 G2) *via* its conserved C-terminal G2BR domain at the LDs surface can initiate lipophagy (Wu et al., 2021). However, a contradictory observation suggests that AUP1 deubiquitination is associated with inducing lipophagy (Wu et al., 2021). Although the upstream signaling pathways for triggering lipophagy are different, the chief pathways involved in lipophagy induction are conserved in most cells. For instance, rapamycin (an MTORC1 inhibitor)-induced lipophagy can enhance the colocalization of an LD marker (BODIPY dye) with a lysosomal marker (LAMP1), and inhibition of AMPK decreases kaempferol-stimulated colocalization of LDs with lipophagosomes and lysosomes (Varshney et al., 2018; Li et al., 2021)

## 5 Xenophagy and virophagy: Selective autophagic elimination of microorganisms

Xenophagy involves the dedicated removal of intracellular pathogenic microorganisms (e.g., viruses and bacteria). Like mitophagy, xenophagy uses several receptors (including SQSTM1, TAX1BP1, CALCOCO2, OPTN and NBR1) to selectively connect the cargo to the phagophore (Sharma et al., 2018; Li et al., 2021). As a subset of xenophagy, virophagy (specifically removal of viruses) has been associated with the elimination of different pathogenic viruses such as human immunodeficiency virus (HIV) (Li et al., 2021) and severe acute respiratory syndrome coronavirus 2 (SARS-CoV-2). Accordingly, the opportunity to target virophagy in its antiviral role against coronavirus disease 2019 (COVID-19) is an attractive therapeutic perspective. The detailed molecular mechanism of virophagy will be discussed in a future study.

COVID-19, an outbreak triggered by the virus SARS-CoV-2, has become a devastating global pandemic with significant impacts on human life and economic systems. Although most infected individuals are asymptomatic, the typical COVID-19 disease presents as mild to severe progressive pneumonia. SARS-CoV-2 infection can affect all the systems of the human body, and emerging data suggest that COVID-19 has pulmonary and extrapulmonary manifestations including hepatic injury subsequently progressing to multiorgan damage and death particularly in elderly patients (Di Sessa et al., 2021).

As with certain other microbes, SARS-CoV-2 subverts the autophagic response to avoid the host cell’s immune response. For example, SARS-CoV-2 infection results in reduced expression of IRF8, which encodes a transcription factor that positively regulates xenophagy (Gupta et al., 2015; Tomić et al., 2021). Furthermore, the viral ORF8 protein interacts with MHC I molecules and the autophagic machinery to downregulate antigen presentation, thus preventing the infected cells from being recognized by T cells (Zhang et al., 2018; 2021). Further studies are needed to fully understand how the virophagy mechanism could be exploited by viruses).

## 6 Transcriptional regulation of selective autophagy

Several selective autophagy-related genes and lysosomal-genes are regulated by microphthalmia (MiT/TFE) subfamily transcription factors (TFs) (Nezich et al., 2015). For example, TFEB regulates not only non-selective autophagy, but also lysosomal biogenesis, mitophagy and lipophagy (Zhang et al., 2018; Kloska et al., 2020; Zhang et al., 2021). MTORC1 acts a major regulator of TFEB transcriptional activity (Al-Bari and Xu, 2020). Under nutrient-rich conditions, TFEB associates with lysosomes where it interacts with the RRAG GTPases and then binds to active MTORC1. TFEB phosphorylation at S211 by MTORC1 generates an interacting site for YWHA/14-3-3, a cytosolic chaperone that retains TFEB in the cytoplasm (Al-Bari and Xu, 2020). Conversely, in response to several stimuli such as pathogen exposure or nutrient starvation, the RRAG GTPases are in an inactive conformation state resulting in MTORC1 inactivation, dissociation of TFEB from both MTORC1 and YWHA/14-3-3 and release from lysosomes. In addition, activated PPP3/calcineurin, a Ca^2+^-dependent phosphatase stimulates dephosphorylation of TFEB and translocation into the nucleus. This event also protects against additional phosphorylation of TFEB by MTORC1 and its binding to YWHA/14-3-3. TFEB nuclear translocation triggers transcription of numerous target genes (Al-Bari and Xu, 2020). In the nucleus, dephosphorylated TFEB interacts selectively with a 10-bp motif (GTCACGTGAC) present in the promoter regions of several genes encoding lysosomal and autophagic proteins. For example, activated TFEB induces autophagy and lysosomal genes by interacting with the coordinated lysosomal expression and regulation/CLEAR element in the regulatory sections of its target genes and enhances the levels of principal controllers of lipid metabolism including PPARGC1A/PGC1α (PPARG coactivator one alpha) and PPARA/PPARα (Kim et al., 2018). TFEB is involved in biogenesis and acidification of lysosomes; autophagosome formation and fusion with lysosomes and mitophagy and mitochondrial biogenesis (Al-Bari and Xu, 2020) as well as regulation of lipophagy (Settembre et al., 2013) by upregulating PPARGC1A expression (Austin and St-Pierre, 2012) under exercise and fasting condition. PPARGC1A co-activates NFE2L2/NRF2 (NFE2 like bZIP transcription factor 2), the main TF for multiple antioxidant proteins.

NFE2L2 has been implicated in maintaining mitochondrial redox homeostasis and biogenesis through the direct upregulation of mitochondrial TFs, and the mitochondrial quality control system through PINK1-PRKN-dependent mitophagy activation. NFE2L2 can also regulate the transcription of TFAM (transcription factor A, mitochondrial). TFAM translocates to the mitochondrial matrix where it stimulates mtDNA replication and mitochondrial gene expression (Tang, 2016). By maintaining a compensatory effect of TFAM, TFEB may launch a positive feedback regulatory loop for maintaining the equilibrium between mitophagy and mitochondrial biogenesis. Like TFEB, NR1H4/FXR (nuclear receptor subfamily one group H member 4) and transcriptional activator CREB (cAMP response element binding protein) regulate lipophagy. Under conditions of nutrient deprivation, CREB triggers lipophagy by upregulating the expression of ATG7, ULK1 and TFEB, but, under fed conditions, NR1H4/FXR suppresses this response (Kloska et al., 2020). Other MiT/TFE family members such as MITF (melanocyte inducing transcription factor) and TFE3 are also required for efficient mitophagy (Nezich et al., 2015). For example, TFE3 regulates autophagy flux, lysosomal biogenesis and hepatic lipid metabolism. By enhancing autophagy flux, TFE3 relieves hepatic steatosis *via* enhancing PPARGC1A-dependent mitochondrial FA β-oxidation. Mechanistically, TFE3 controls PPARGC1A by interacting with the promoter region of its cognate gene (Xiong et al., 2016; Li et al., 2021). Lipophagy and lipolysis are also regulated by TFE3. TFE3 induces lipophagy and alleviates liver steatosis in mice (Ploumi et al., 2017). As with TFEB, TFE3 stimulates the mRNA level of genes encoding PPARGC1A and PPARA that modify mitochondrial FA β-oxidation (Xiong et al., 2016). Furthermore, TFE3 insufficiency results in altered mitochondrial morphology and function (Pastore et al., 2017), whereas TFEB overexpression in the absence of TFE3 improves the metabolic outcome (Ni et al., 2013) due to compensatory effects.

SIRT (sirtuin) proteins are nicotinamide adenine dinucleotide (NAD)-dependent deacetylases that have several actions regarding mitochondrial protection in response to various stresses, and FA composition. For example, SIRT3 promotes BNIP3-mediated mitophagy *via* inducing the MAPK/ERK-CREB signaling pathway. Moreover, SIRT3 can restrict HBV transcription and replication, limiting inflammation-mediated liver damage during HBV infection (Li et al., 2018). FOXO (forkhead box O) family member TFs play important roles in mitochondrial remodeling processes. For instance, FOXO3 directly upregulates the expression of PINK1 that controls mitochondrial remodeling (Ploumi et al., 2017). Interestingly caloric restriction induces SIRT1-dependent mitophagy and attenuates hypoxia-associated mitochondrial damage. SIRT1 deacetylates FOXO3 which induces mitophagy by promoting expression of BNIP3 (Ploumi et al., 2017). Acute ethanol exposure inhibits AKT/protein kinase B and causes FOXO3/FOXO3A dephosphorylation and subsequent nuclear translocation. In the nucleus, FOXO3 interacts with the promoter regions and enhances the levels of *ATG5*, *ATG7*, *BECN1* and *ULK1* resulting in induction of autophagy in hepatocytes (Ni et al., 2013). Similarly, FOXO1 promotes mitophagy through regulating the transcription of *PINK1* and *LC3* in a ROS-dependent manner (Wang D. et al., 2020). In addition, FOXO1 facilitates the alteration of mitochondrial dynamics through the DNM1L pathway. Hepatic lipophagy is also controlled by ATG14-mediated FOXO family TFs and circadian rhythms (Czaja et al., 2013). Under conditions of nutrient deprivation, FOXO1 and TFEB are upregulated which activates the lipolytic pathway by stimulating the level of LIPA. Lysosomal stress conditions activated by atherogenic lipids promotes nuclear TFEB translocation and causes upregulation of LIPA and biogenesis of lysosomes (Kloska et al., 2020). Furthermore, mitochondrial localized STAT1 (signal transducer and activator of transcription 1) modulates mitophagy (Patoli et al., 2020).

Lipophagy also controls lipid catabolism by contributing to the regulation of PPARA action *via* NCOR1 (nuclear receptor corepressor 1) degradation. In response to fasting, autophagic NCOR1 degradation allows PPARA induction for promoting FA β-oxidation. Defective autophagy causes NCOR1 accumulation and inhibition of PPARA activity resulting in impaired β-oxidation (Saito et al., 2019; Kloska et al., 2020).

## 7 Roles of mitophagy and lipophagy in hepatic physiology and pathophysiology

The liver is a unique organ based on its functional properties and regenerative capability because liver cells contain a large mitochondrial mass as well as supply glucose for the entire body and store lipids. The liver also acts as an immune organ in the body because anatomically it receives the portal blood supply from the gut and encounters the incoming challenge of orally swallowed intestinal bacteria and their metabolic antigens. Finally, hepatocytes are the primary sites of various liver-trophic viral infections that are among the most common infections in the world (Czaja et al., 2013). Moreover, the liver is a vital organ for lipid storage and subsequent mobilization by lipogenesis and the main site for the packaging, redistribution, and processing of FAs. Accordingly, reduced hepatic lipid catabolism is tightly interrelated with various liver diseases. Accumulating evidence indicates that mitophagy and lipophagy potentially play a vital role in the control of liver homeostasis (Czaja et al., 2013; Eid et al., 2016). Improper regulation of these selective types of autophagy is thought to contribute to the pathogenesis of several diseases including metabolic syndrome and liver injury (Ke, 2020). Now, it is clear that deregulation of mitophagy and aberrant lipophagy contribute to the progression of liver-associated diseases including non-alcoholic fatty liver disease (NAFLD), alcoholic fatty liver disease (AFLD), drug-induced liver injury (DILI), hepatic ischemia-reperfusion (I/R) injury, viral hepatitis and liver cancer (Eid et al., 2013a; Eid et al., 2013b; Ma et al., 2020) (detailed in Supplementary Table S1). This comprehensive understanding of the mechanistic insights will provide the background to the prospects of identifying novel selective autophagy-related therapeutic targets for the development of an efficient strategy for the treatment of liver diseases.

### 7.1 Non-alcoholic fatty liver disease (NAFLD) and alcoholic fatty liver disease (AFLD)

The pathogenesis of NAFLD originates from abnormal liver lipid metabolism, including enhanced lipogenesis, raised FFA uptake and lipid accumulation in hepatocytes (Czaja et al., 2013). NAFLD covers a spectrum of hepatic abnormalities that begins with hepatic steatosis and its progression to inflammatory hepatocellular injury known as non-alcoholic steatohepatitis (NASH) that subsequently develops into liver fibrosis, cirrhosis, and hepatocellular carcinoma (HCC) (Czaja et al., 2013; Ma et al., 2020; Niture et al., 2021). Mitophagy plays a central role in NAFLD pathophysiology (Niture et al., 2021) because healthy mitochondria have critical functions in lipid metabolism, and decreased mitochondrial action promotes NAFLD (Ma et al., 2020). The lipotoxicity within the liver activates a series of mitochondrial dysfunctional (failure of mitochondria to function normally) events including excessive oxidative stress or inflammation (Mansouri et al., 2018; Doblado et al., 2021). Although the emerging role of lipophagy in NAFLD is still not firmly established, activation of lipophagy is apparent during NAFLD progression (Czaja et al., 2013). APOB (apolipoprotein B)-oriented LDs (Ohsaki et al., 2006) and bortezomib-induced Mallory Denk bodies (MDBs) in liver cells (Strnad et al., 2008; Harada et al., 2008; Harada, 2010) can be eliminated by lipophagy, suggesting that lipophagy can protect against NAFLD development. Thus, impaired mitophagy and lipophagy can contribute to the development of NAFLD and subsequent HCC. Metabolic-associated fatty liver disease (MAFLD) patients have a higher risk of SARS-CoV-2 infection and increased liver dysfunction in comparison to patients without MAFLD. Dysregulated hepatic immunity in NAFLD patients can participate in COVID-19 pathogenesis because immune cells (e.g., Kupffer cells) can produce active cytokines and aggravate or contribute to the cytokine storm (Di Sessa et al., 2021).

Similar to NAFLD, AFLD caused by chronic alcohol abuse has a wide spectrum of pathogenesis such as steatosis, alcoholic hepatitis and fibrosis which can develop into cirrhosis and even HCC (Czaja et al., 2013; Eid et al., 2013a; Eid et al., 2013b; Niture et al., 2021). Ethanol metabolism through ADH (alcohol dehydrogenase) and/or CYP2E1 (cytochrome P450 family two subfamily E member 1) causes an excess amount of acetaldehyde, an enhanced NADH:NAD^+^ ratio and an increased oxidative stress that induce autophagy (Czaja et al., 2013; Niture et al., 2021). Excessive oxidative stress enhances lipid accumulation and mitochondrial damage that contribute to liver injury (Khambu et al., 2017). Excessive alcohol consumption can cause defective lipid export from liver tissues, accumulation of LDs in the liver and dysregulation of mitochondrial homeostasis (Niture et al., 2021). Chronic alcohol use can cause failure of the hepatic protein degradation system (Khambu et al., 2017) by causing defects in both proteasome and lysosomal action, resulting in the formation of MDBs which are composed of cytosolic inclusion bodies enriched with ubiquitin, SQSTM1 and cytoskeletal intermediate filament proteins such as hepatocyte KRT8 (keratin 8) (Niture et al., 2021). Inhibition of MTOR significantly reduces the number of MDBs and promotes clearance of MDBs in proteasome-inhibitor treated KRT8 transgenic mice (Czaja et al., 2013; Khambu et al., 2017). Autophagy triggered by ethanol seems to selectively remove damaged mitochondria (*via* mitophagy) and LDs (*via* lipophagy) that accumulate in liver cells (Czaja et al., 2013; Lu et al., 2015; Williams et al., 2015; Eid et al., 2016). Alcohol consumption suppresses mitochondrial respiratory complex protein synthesis resulting in mitochondrial damage (Niture et al., 2021) and this damage-induced mitophagy predominantly exists in AFLD (Khambu et al., 2017). Also, chronic ethanol consumption causes a reduction of oxidative phosphorylation, enhancement of oxidative damage to mitochondrial DNA (mtDNA) causing strand breakage and impaired mitophagy resulting in AFLD pathogenesis (Khambu et al., 2017; Niture et al., 2021). There are no published studies looking exclusively at the outcomes of AFLD patients with COVID-19. However, the proportion of patients with AFLD have increased the risk of mortality in the early part of the pandemic compared to the pre-pandemic era (Elhence et al., 2021).

### 7.2 Hepatocellular cancer (HCC)

Many human diseases including liver cancer are associated with mutations in core components of the mitophagy machinery and disrupted mitochondrial dynamics. Autophagy, mitophagy (Wang Y. et al., 2015; Wang Y. et al., 2020) and lipophagy (Amaddeo et al., 2021) have ambiguous functions in cancer, in early stages functioning as tumor suppressors but in established stages promoting progression by supporting the metabolic demand. The controversial dual role of selective autophagy has been studied in a wide variety of cancers, emphasizing its importance in carcinogenesis (Czaja et al., 2013; Niture et al., 2021). Lipophagy also participates in the utilization of stored LDs, allowing cancer cells to access this latent supply of energy for their growth (Kounakis et al., 2019). During lipophagy, CEBPA/C/EBPα (CCAAT enhancer binding protein alpha) is upregulated in HCC patients, promoting resistance to nutrient deprivation and contributing to carcinogenesis (Lu et al., 2015). As described in earlier sections, weakened mitophagy aggravates both NAFLD and AFLD and may participate in the progression of HCC. The presence of HCC is involved in an enhanced risk of COVID-19-related mortality (Amaddeo et al., 2021; Elhence et al., 2021; Kim and Lemasters, 2021). Thus, targeted therapy based on mitophagy and lipophagy is of current interest as a potential strategy for the treatment of cancer. It is worth mentioning that HCC can be initiated by excessive alcohol consumption *via* various mechanisms such as oxidative stress, mitochondrial damage, and steatosis and fibrosis (Eid et al., 2013a; Eid et al.2013b; Eid et al., 2016).

### 7.3 Drug-induced liver injury (DILI)

The most common cause of acute liver failure in the United States is associated with DILI. Drugs causing DILI include antivirals, antibiotics, and immunosuppressive drugs, and many others have been connected to liver injury. Acetaminophen (APAP also known as paracetamol in the United Kingdom) toxicity, idiosyncratic or dose-independent DILI occurs relatively frequently (Williams and Ding, 2020). Mitochondria play a key role in controlling cell death and DILI (Ma et al., 2020). For example, diclofenac, a non-steroidal anti-inflammatory drug (NSAID) for the treatment of rheumatic diseases may be involved in drug-induced mitochondrial injury and dysfunction (Ramachandran et al., 2018). Thus, co-treatment with the AMPK activator 5-aminoimidazole-4-carboxamide ribonucleotide (AICAR) inhibits diclofenac-induced mitochondrial depolarization and hepatotoxicity (Kang et al., 2016). Since rapamycin treatment is not able to inhibit diclofenac-induced mitochondrial injury in hepatocytes, the MTOR-dependent autophagy pathway may not be involved in this preventive mechanism.

### 7.4 Viral hepatitis

**FIGURE 5:**
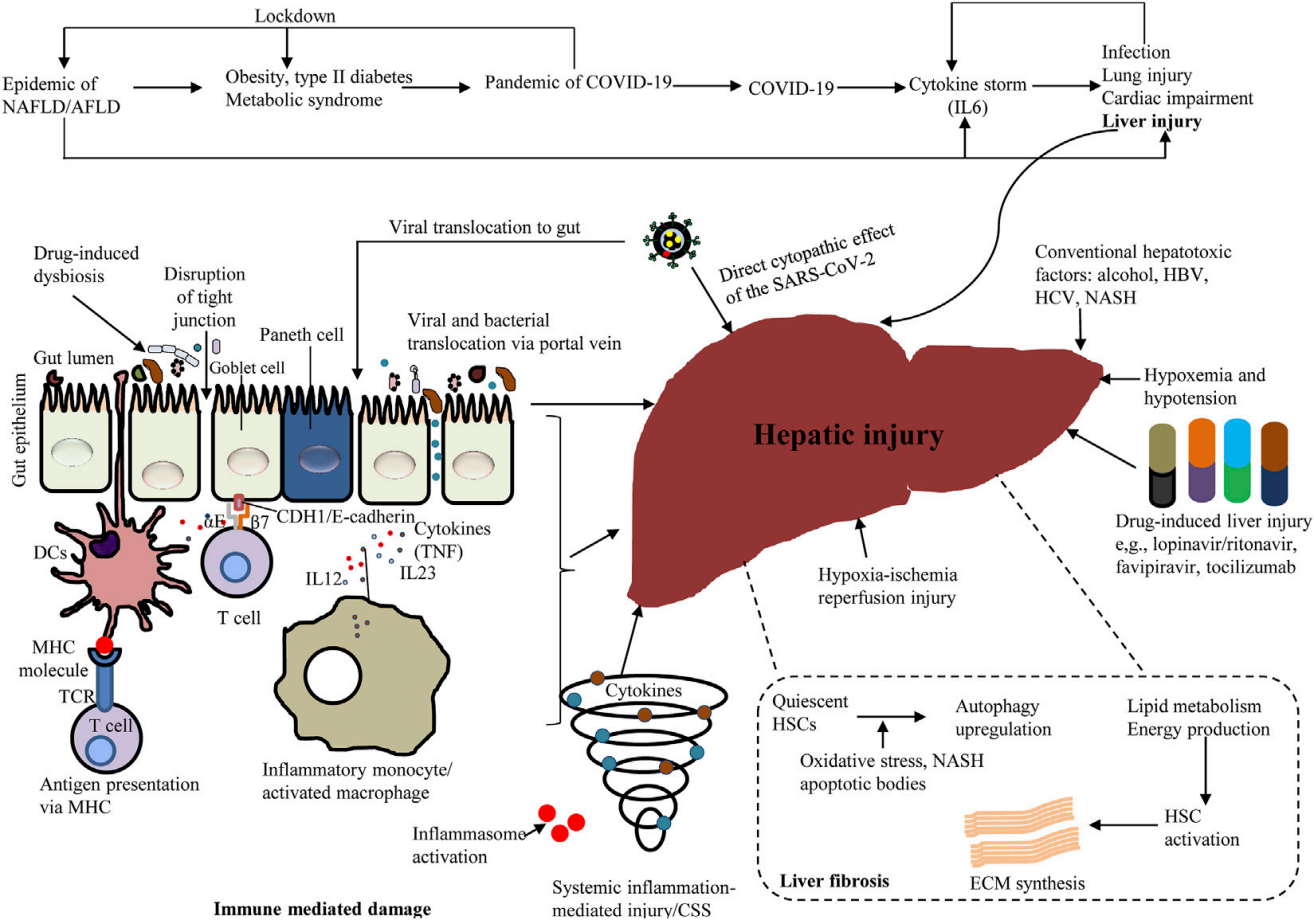
A schema showing the various diseases inducing hepatic injury, including COVID-19.

Research on autophagic action in liver immunity is inadequate and usually limited to hepatitis viruses. Numerous infectious microorganisms suppress autophagy and dull the immune response; or otherwise, redirect autophagy for promicrobial action (Czaja et al., 2013). Viruses that target the liver, including HBV, hepatitis C virus (HCV) and dengue virus (DENV) as well as the current life-threatening SARS-CoV-2, all commandeer autophagy for proviral purposes. Autophagy appears to promote HBV replication, as autophagy suppression decreases HBV replication in cells and prevents HBV envelopment (Czaja et al., 2013). Several studies have shown that HCV, a small enveloped RNA virus (Czaja et al., 2013; Ma et al., 2020) can influence autophagy, although it is unclear whether HCV can enhance autophagy. Interestingly, HCV mRNA and protein levels in the host cell remain unchanged in ATG7^−/−^ and BECN1^−/−^ HCV-infected cells (Tanida et al., 2009). However, these proteins are still required for autophagosome maturation and promotion of HCV RNA replication (Czaja et al., 2013). HCV-infected cells accumulate LDs that play an essential role in the assembly of viral particles. Interestingly, autophagy can counteract the changes in lipid metabolism triggered by HCV (Vescovo et al., 2019). Patients with HCV-related and chronic liver disease (CLD) are at greater risk for getting SARS-CoV-2 infections (Elhence et al., 2021). Although hepatic DENV infection does not causes hepatitis, DENV is a key global health problem causing the clinical outcomes of dengue fever. DENV infection occurs in a variety of cells including hepatocytes, and autophagy plays a proviral role in DENV replication in this cell type. DENV-mediated lipophagy triggers lipid metabolism. DENV infection increases autophagosome formation and enhances the association of autophagosomes with LDs (Czaja et al., 2013). Figure 5 demonstrates mechanisms of hepatic injury by various diseases including COVID-19. Supplementary Table S1 summarizes the various roles of selective autophagy (mitophagy and lipophagy) in liver physiology and pathophysiology.

## 8 The interplay of selective autophagy pathways in hepatic pathology

The interplay between different types of selective autophagy is not well studied. A recent study found that the *Mycobacterium* *bovis* utilizes host mitophagy to suppress host xenophagy to enhance its intracellular survival (Song et al., 2021). Consequently, it would be necessary to determine if this applies to liver diseases caused by viruses, such as hepatitis C and SARS-CoV-2. Furthermore, activation of PRKN-mediated mitophagy may mitigate hepatic steatosis by activation of lipophagy in animal models of binge ethanol exposure (Williams et al., 2015; Eid et al., 2016). An interesting review article concluded that some autophagy blockers such as chloroquine and hydroxychloroquine may inhibit SARS-CoV-2 replication *via* inhibition of lysosomal fusion with autophagosomes (Maity and Saha, 2021). There is growing evidence that excessive accumulation of LDs in various organs of obese people speeds up the replication of SARS-CoV-2 and reduces its elimination through various mechanisms related to lipid overload (Dias et al., 2020; Goossens et al., 2020; Rebello et al., 2020; Yan et al., 2021). Consequently, stimulating LD clearance by lipophagy using drugs or natural products may reduce virus replication and increase its clearance by virophagy. Conversely, a recent study found that flaviviruses exploit the LD protein AUP1 to trigger lipophagy and drive virus production in DENV-infected cells (Zhang et al., 2018). Further studies are needed to understand the molecular mechanisms controlling the crosstalk between the various types of selective autophagy and the implications for health and liver diseases.

## 9 Conclusions and future directions

Autophagy is vital for regulating normal liver physiology. In the current review, we have focused on how selective autophagy (mainly mitophagy and lipophagy) affects liver pathologies such as NAFLD, AFLD, and HCC progression and severity. Defective autophagy is insufficient to degrade accumulated LDs in the liver, causing hepatic steatosis, a primary episode in NAFLD or AFLD. However, many questions remain before we can understand the roles of selective autophagy in these diseased conditions. The fact remains that there are no exact protocols to evaluate the precise levels of autophagy dynamics in patients. Because the dynamics and stages of autophagy vary greatly during the progression of these diseases, a selective precise method is necessary to monitor and assess the type and magnitude of autophagy. Thus, development of appropriate methods, biomarkers of autophagy impairment and biomarkers for the *in vivo* spectrum of liver diseases are also significant hurdles in the discovery of autophagy-targeting strategies. In addition, detection, and measurement of some metabolites in the blood, saliva or urine that are secreted *via* autophagy-dependent pathways may be useful. Promoting active autophagy and restoring defective autophagy by repurposing drugs or natural products would be hugely beneficial for the treatment of hepatic diseases. Artificial intelligence technology can also be used to enhance drug discovery. Analyses of autophagosome numbers, or levels of autophagy markers such as ATGs, LC3-II or BECN1 are not sufficient to evaluate autophagic action and monitoring of UPS-mediated degradation should be considered. As autophagy can promote cell viability in extreme stress, an appropriate assessment of autophagy level in patients with liver disease is essential for clinical applications.

Interestingly, HBV, HCV, DENV and SARS-CoV-2 have evolved unique mechanisms to augment autophagic action for their own propagation in the liver. Mitophagy seems to be a vital cellular process that can promote health by maintaining mitochondrial turnover and integrity, and thus maintain physiological liver function. Accumulating evidence implies that mitophagy and lipophagy defend liver cells from damage and act as protective mechanism against the development of liver diseases such as DILI. Thus, boosting hepatic mitophagy and lipophagy appears to be an auspicious tactic in the development of novel therapies for liver diseases. However, the precise roles of mitophagy and lipophagy are controversial, and disrupting these processes can exacerbate liver pathogenesis (e.g., HCC development and its progression), indicating that appropriate control of selective autophagy must be coordinately managed to relieve liver diseases. In addition, hepatic parenchymal and non-parenchymal cells have different lipophagic responses to various stressors, and liver cell-specific lipophagy molecules are not well identified. Furthermore, there are huge inconsistencies among different experimental and clinical studies; and most conclusions on selective autophagy (mitophagy)-based liver pathology are experimental model- and stage of disease-dependent, hindering the comprehensive understanding of the roles of selective autophagy in liver diseases. Thus, advanced studies are necessary to understand the comprehensive function(s) of selective autophagy in different stages of liver diseases and to develop this understanding sufficiently to produce clinically significant therapeutic strategies.
