# Supplementary material for: Emerging mechanistic insights of selective autophagy in hepatic diseases
Source: Front Pharmacol. 2023 Mar 16;14:1149809. doi: 10.3389/fphar.2023.1149809 (PMC10060854; doi:10.3389/fphar.2023.1149809)
Supplement: Supplementary file 1 [file DataSheet1.pdf]

Supplementary Table 1 and Related References and Supplementary Fig.1 legend

Table 1 S

Actions of selective autophagy (mitophagy and lipophagy) in liver physiology and pathophysiology.

Mechanistic events	Experimental model with references
Defective autophagy in NAFLD steatosis/NASH	
Hepatic steatosis suppresses autophagic proteolysis via alteration of lysosomal acidification and proteinase activity of cathepsin B/L	Obese (ob/ob) mice [Inami et al., 2011; Niture et al., 2021]
Changes in membrane lipid composition reduce autophagosome-lysosomal fusion up to 70%	treatment with methyl-β-cyclodextrin (MBCD) or male B6 mice fed with HFD challenge [Koga et al.2010; Niture et al.2021]
IR and hyperinsulinemia lead to suppress autophagy via FOXO1-mediated downregulation of key autophagy genes. IR also suppresses mitophagy *in vitro*	HFD fed C57BL/6 mice [Liu et al.2009; Niture et al.2021]
Over-expression of Rubicon impairs autophagy in liver	HFD fed male C57BL/6 mice or steatotic patient samples [Tanaka et al.2016; Niture et al.2021]
Inhibition of SERCA leads to aggregate in cytosolic Ca^{2+} levels and reduced autophagic flux	HFD induced obese mice or hepatocytes treated with SFA (e.g., PA) [Park et al.2014]
Activated PP2A leads to inhibit autophagic lipid catabolism that contributes to liver steatosis	Hepatocytes treated with methionine and SAM [Zubiete-Franco et al., 2016]
Decreased expressions of lipid-metabolizing enzymes (CPT1A, PPARA, ACAT1, ACADVL) and reduced autophagic flux reduces lipid oxidation and increases mitochondria swelling in the liver	Genetic ablation of *pld1*$^{-/-}$ (phospholipase D1) mice [Hur et al., 2016]
Increase SIRT3 (a negative regulator of autophagy), inactivate AMPK1 and overactivate MTOR leading to impaired autophagy	Mice fed with an SFA(PA)-rich HFD [Li et al., 2017]
Activation of TBK1 leads to induce impaired autophagy, aggregate ubiquitinated proteins resulting in lipotoxicity in hepatocytes	Hepatocytes treated with SFAs (PA and SA)[Cho et al.2018]
Upregulation of SQSTM1, LC3-II and accumulation of autophagosomes, suggests a defective autophagic flux.	Mice fed with HFD or MCD;<br>Huh7 cells treated with PA [Gonzalez-Rodriguez et al., 2014]

Activation of IBTKα, a member of UPR results in induction of autophagy and activation of NFKB triggering hepatocyte lipotoxicity	SFA (PA) treated HepG2 cells [Willy et al., 2017]
Hepatic steatosis induces defective autophagy in the liver and inhibits autophagic proteolysis	In human NAFLD, chronic hepatitis B and chronic hepatitis C patient samples [Fukuo et al., 2014]
Defective autophagy in AFLD	
mTOR activation and a decrease levels of TFEB-mediated lysosomal gene expression lead to defective autophagy	Chronic feeding plus acute binge alcohol ("Gao-binge") in mice [Chao et al., 2018]
Activation of ACAC/ACC activity and increased malonyl CoA content in liver tissues suppress autophagy.	H4IIEC3 hepatoma cells, chronic Lieber-DeCarli alcohol in C57BL/6J mouse model [You et al., 2004]
Downregulation of BECN1 and ATG5 and upregulation of SQSTM1 in liver tissues impair hepatocellular autophagy	Male Wistar rats fed a Lieber-DeCarli diet alcohol (5%) for chronic exposure [Menk et al., 2018]
Inactivation of RAB7 and DNM2 result in impaired lipophagy.	Alcohol-containing fed Lieber-DeCarli diet in male Wistar rats [Bao et al., 2014; Rasineni et al., 2017]
Activities of LAL decreases in hepatocytes	Chronic ethanol exposure in rats [Kharbanda et al., 1996; Schulze et al., 2017]
Defective autophagy in HCC	
Higher expressions of SQSTM1 and GPC3 (glypican 3; a tumor marker for HCC) indicate defective autophagy liver cancer.	Patients with HCC cells with HCV infection [Bao et al., 2014]
Impaired autophagy by increasing oxidative stress leads to start hepatocarcinogenesis.	Liver-specific *atg5*$^{-/-}$ mice [Tian et al., 2015]
HuR increased autophagy mRNA expressions leads to impaired autophagy in HCC cells.	Hep3B, Huh7 and THLE-3 cells [Ji et al., 2019]
Impaired autophagy and increased development of liver tumors	Mosaic deletion of *Atg5* and liver-specific ATG7-deficient mice [Takamura et al., 2011]
Defective autophagy increases the frequency of spontaneous tumors.	Targeted deletion of BECN1 in embryonic stem (ES) cells or mice [Yue et al., 2003]
Actions of mitophagy in liver physiology	
The distorted mitochondria associated with α1-ATD-mediated chronic liver diseases	Liver specimens from patients with an α1-ATD; liver samples of SERPINA1/α1-AT Z variant Tg mice [Perlmutter, 2002; Teckman et al., 2004]
Age-dependent loss of mitophagy	Aged rat liver tissues [Donati et al., 2006; Cavallini et al., 2007]
Impaired autophagic clearance of damaged mitochondria	Liver tissue specimens of *atg7*$^{-/-}$ mice [Komatsu et al., 2005, Ke 2020]
Control the intracellular inclusion body formation	*atg7*$^{-/-}$ *sqstm1*$^{-/-}$ mice [Komatsu et al., 2007; Czaja et al., 2013]
Liver autophagy regulates blood glucose and amino acid levels	Liver-specific *atg7*$^{-/-}$ mice [Ezaki et al., 2011, Czaja et al., 2013]

Starvation-induced mitophagic degradation	Liver tissue specimens of GFP-LC3 Tg mice [Kim and Lemasters, 2011; Ke 2020]
Loss of BNIP3-dependent mitophagy decrease mitochondrial turnover	Liver tissues and primary hepatocytes from *bnip3* KO mice [Glick et al., 2012]
Mitophagic degradation and aging provoke an impairment in Parkin-mediated mitophagy	Rat liver tissues after I/R [Kim et al., 2008; Li et al.2018; Ke et al.2020]
Starvation-induced mitophagic degradation	Liver specimens from acute liver damage patient [Kheloufi et al., 2014]
Hepatocyte remodeling by mitophagy	Primary rat hepatocytes [Rodriguez-Enriquez et al., 2009]
Parkin-dependent mitophagy protects against ethanol-induced liver injury by	Liver specimens from GFP-LC3 Tg mice and PMH (ethanol treatment) [Eid et al., 2015; Williams et al., 2015b; Williams and Ding 2015a]
Mitophagy protected against efavirenz (antiretroviral drug)-induced hepatic injury	Hepatocytes, Hep3B cell line and PMH (treated with efavirenz) [Apostolova et al., 2011a; Apostolova et al., 2011b]
Mitophagy protects against Cd-induced hepatotoxicity	The human normal liver cell line, L02 (Cd treated) [Pi et al., 2013]
Protection against APAP-induced liver injury by mitophagy through AMPK activation	Liver tissues from GFP-LC3 Tg mice (APAP treatment) [Gordon, 1973; Shan et al., 2019; Sun et al., 2019]
Protection against I/R-induced hepatic injury by SIRT1 and PRKN-dependent mitophagy	Liver specimens from human patients, PMH and liver tissues of I/R-treated *sirt1*$^{-/-}$ mice [Rodriguez-Enriquez et al., 2009; Biel et al., 2016; Chun et al., 2018; Hong and Lee, 2018; Ning et al., 2018]
Protection against I/R-induced liver injury by HO-1-induced mitophagy through PGAM5 activation	Liver tissue specimens of I/R-treated mice [Hong and Lee, 2018]
Protected against I/R-induced liver injury by *Mir330-3p*-induced mitophagy via upregulation of PGAM5 expression	Liver tissue specimens of I/R-treated mice and liver cell line, L02 [Sun et al., 2019]
Actions of mitophagy in liver injury: steatosis and fatty liver diseases	
Mitochondrial dysfunction is associated with ALFD and NAFLD	Liver specimens with ALFD and NAFLD patients [Gordon, 1973; Ke 2020]
Defense against fatty liver by PINK1-PRKN-dependent mitophagy	Rat liver specimens (ethanol treatment) [Eid et at. 2013; Wiliam et al.2015; Eid et al. 2016a,b]
Mitochondrial dysfunction is associated with NAFLD	Liver specimens from patients with NAFLD [Caldwell et al., 1999; Ke et al.2020]
Defense against NAFLD by PRKN-dependent mitophagy	Liver tissues from *alcat1* KO mice fed with a HFD [Wang et al., 2015]
Suppression of NAFLD development by TH-induced mitophagy	HepG2 cells and mouse liver tissues fed with a MCD [Sinha and Yen, 2016]

Initiation of DRAM-mediated mitophagy in the development of NAFLD	HepG2 cells treated with OA [Pang et al., 2018]
Megamitochondria by defective PRKN-independent mitophagy in fatty liver	Liver tissues from liver-specific *dnm1l*$^{-/-}$, *opa1*$^{-/-}$ mice and *dnm1l*$^{-/-}$ *opa1*$^{-/-}$ mice with MCD feeding [Yamada et al., 2018]
Increased mitochondrial protein degradation by activated mitophagy	Liver tissues from LDLR KO with a WD feeding [Lee et al., 2018]
Inflammasome activation by inhibition of mitophagy	Mouse liver tissues fed a HFCD and rat primary hepatocyte treated with PA [Zhang et al., 2019]
Actions of mitophagy in liver cancer	
Suppression of hepatoma cell growth and liver tumor by ConA-activated BNIP3-dependent mitophagy	BALB/c hepatoma cell line ML-1; liver specimens from NOD/SCID mice treated with ConA [Chang et al. 2007; Lei and Chang, 2007]
Activation of Dox-activated cell death of hepatoma cells	HepG2 cells treated with Dox (adriamycin) [Qian and Yang 2009]
Improvement of Dox-activated cell death of HepG2 cells by curcumin	HepG2 cells treated with Dox and curcumin [Qian et 2011]
Cytotoxicity of sorafenib is amplified in hepatoma cells by melatonin-activated mitophagy	HepG2, Hep3B and Huh7 cells treated with melatonin and sorafenib [Prieto-Domínguez et al.2016]
Cell death of hepatoma cells is triggered by inducing DRAM-dependent mitophagy	HepG2, Hep3B and Huh7 cells [Liu et al., 2014]
Inhibition of initiation of HCC by FUNDC1-activated mitophagy through suppression of inflammasome	Liver tissue specimens from HCC patients and liver tissues from liver-specific *fundc1* KO mice [Li et al., 2019]
Elevation of HCC cell viability by upregulated DNM1L and decreased MFN1 levels	Liver tissue specimens of HCC patients and mouse xenograft models, Bel7402 and SMMC7721 cell lines [Huang et al., 2016]
Preservation of the stemness of CSCs by activating NANOG and initiation of mitophagy	HepG2, Hep3B and Huh7 cells [Liu et al., 2017]
Actions of mitophagy in viral hepatitis	
PINK1-PRKN-dependent mitophagy elevates HCV replication, and HCV-induced mitophagy reduces infected cell apoptosis	Huh7.5.1 cells with HCV infection [Kim et al 2013b; Kim et al., 2014]
HCV-activated mitophagy degrades depolarized mitochondria	Huh7.5.1 cells transfected with HCV NS5A [Jassey et al., 2019]
A continual HCV infection causes mitochondrial damage by inhibiting mitophagy	Huh7 cells with HCV infection and Liver tissues of HCV Tg mice [Hara et al., 2014]
HBV-induced PINK1-PRKN-mediated mitophagy protects infected cells from apoptosis	Huh7 cells transfected with HBV [Kim et al., 2013a]
Nutrient deprivation-induced PINK1-PRKN-dependent mitophagy is augmented by regulatory protein, HBx	HepG2 cells and SMMC-7721 cells (transfection of HBx) [Kim et al., 2013b]

Actions of mitophagy in other liver diseases	
Melatonin-induced mitophagy protects against CCl_4-induced liver fibrosis	CCl_4 treated rat liver tissue specimens [Kang et al., 2016]
CCl_4 activates of PINK1-PRKN-dependent mitophagy in Kupffer cells but TIMD4/TIM-4 suppresses it	CCl_4 treated mouse liver tissue specimens [Wu et al 2020]
Fine particulate matter (PM2.5) activates HSCs and causes liver fibrosis; and inhibition of mitophagy alleviates the fibrosis	LX-2 cells and primary HSCs [Qiu et al., 2019]
Defective mitophagy promotes inflammasome activation in the HSC model	Liver specimens from patients with acute liver failure and mice treated with LPS and LX-2 cells treated with H_2O_2, LPS, NAC or FCCP [Tian et al., 2018]
NR4A1-PRKDC-TP53 axis acts as a signaling pathway for AFLD pathogenesis	Hepatocytes from *nr4a1* KO mice, and liver-specific *prkdc* KO mice (ethanol treatment) [Zhou et al., 2019]
Defective mitophagy increases lipogenesis via upregulation of lipogenic enzymes	Liver tissues from *bnip3* KO mice and PMH from *bnip3*-null mice [Glick et al., 2012]
TH-activated mitophagy increases FA β-oxidation through inducing CPT1α expression	Liver specimens from *thr* KO mice and HepG2 cells [Singh et al., 2018]
HFD-fed REDD1 KO mice increase CPT1A, BNIP3 and PRKN expression in the livers	Liver specimens from *ddit4/redd1* KO mice treated with HFD [Dumas et al., 2020]
Insulin resistance (IR) inhibits mitophagy	Liver specimens from B6 mice with HFD and PMH [Liu et al., 2009]
Any defect in PRKN-dependent mitophagy does not change in obesity and IR	Liver specimens from *prkn* KO mice and PMH treated with HFD [Costa et al., 2016; Edmunds et al., 2019]
Loss of FUNDC1-mediated mitochondrial turnover induce adipose tissue-associated macrophage infiltration	Liver specimens from *fundc1* KO mice and PMH treated with HFD [Wu et al., 2019]

Supplementary References (related to supplementary table 1 and supplementary Fig.1 legend)

Al-Bari, M.A.A., Ito Y, Ahmed S, Radwan N, Ahmed HS, Eid N, 2021. Targeting Autophagy with Natural Products as a Potential Therapeutic Approach for Cancer. Int J Mol Sci.10;22 (18):9807

Apostolova, N.; Gomez-Sucerquia, L. J.; Gortat, A.; Blas-Garcia, A.; Esplugues, J. V., Autophagy as a rescue mechanism in efavirenz-induced mitochondrial dysfunction: a lesson from hepatic cells. Autophagy 2011a, 7, (11), 1402-4.

Apostolova, N.; Gomez-Sucerquia, L. J.; Gortat, A.; Blas-Garcia, A.; Esplugues, J. V., Compromising mitochondrial function with the antiretroviral drug efavirenz induces cell survival-promoting autophagy. Hepatology 2011b, 54, (3), 1009-19.

Bao, L.; Chandra, P. K.; Moroz, K.; Zhang, X.; Thung, S. N.; Wu, T.; Dash, S., Impaired autophagy response in human hepatocellular carcinoma. Exp Mol Pathol 2014, 96, (2), 149-54.

Biel, T. G.; Lee, S.; Flores-Toro, J. A.; Dean, J. W.; Go, K. L.; Lee, M. H.; Law, B. K.; Law, M. E.; Dunn, W. A., Jr.; Zendejas, I.; Behrns, K. E.; Kim, J. S., Sirtuin 1 suppresses mitochondrial dysfunction of ischemic mouse livers in a mitofusin 2-dependent manner. Cell Death Differ 2016, 23, (2), 279-90.

Caldwell, S. H.; Swerdlow, R. H.; Khan, E. M.; Iezzoni, J. C.; Hespenheide, E. E.; Parks, J. K.; Parker, W. D., Jr., Mitochondrial abnormalities in non-alcoholic steatohepatitis. J Hepatol 1999, 31, (3), 430-4.

Cavallini, G.; Donati, A.; Taddei, M.; Bergamini, E., Evidence for selective mitochondrial autophagy and failure in aging. Autophagy 2007, 3, (1), 26-7.

Chang, C. P.; Yang, M. C.; Liu, H. S.; Lin, Y. S.; Lei, H. Y., Concanavalin A induces autophagy in hepatoma cells and has a therapeutic effect in a murine in situ hepatoma model. Hepatology 2007, 45, (2), 286-96.

Chao, X.; Wang, S.; Zhao, K.; Li, Y.; Williams, J. A.; Li, T.; Chavan, H.; Krishnamurthy, P.; He, X. C.; Li, L.; Ballabio, A.; Ni, H. M.; Ding, W. X., Impaired TFEB-Mediated Lysosome Biogenesis and Autophagy Promote Chronic Ethanol-Induced Liver Injury and Steatosis in Mice. Gastroenterology 2018, 155, (3), 865-879 e12.

Cho, C. S.; Park, H. W.; Ho, A.; Semple, I. A.; Kim, B.; Jang, I.; Park, H rt al. Lipotoxicity induces hepatic protein inclusions through TANK binding kinase 1-mediated p62/sequestosome 1 phosphorylation. Hepatology 2018, 68, (4), 1331-1346.

Chun, S. K.; Lee, S.; Flores-Toro, J.; U, R. Y.; Yang, M. J.; Go, K. L.; Biel, T. G.; Miney, C. E.; Pierre Louis, S.; Law, B. K.; Law, M. E.; Thomas, E. M.; Behrns, K. E.; Leeuwenburgh, C.; Kim, J. S., Loss of sirtuin 1 and mitofusin 2 contributes to enhanced ischemia/reperfusion injury in aged livers. Aging Cell 2018, 17, (4), e12761.

Costa, D. K.; Huckestein, B. R.; Edmunds, L. R.; Petersen, M. C.; Nasiri, A.; Butrico, G. M.; Abulizi, A.; Harmon, D. B.; Lu, C.; Mantell, B. S.; Hartman, D. J.; Camporez, J. P.; O'Doherty, R. M.; Cline, G. W.; Shulman, G. I.; Jurczak, M. J., Reduced intestinal lipid absorption and body weight-independent improvements in insulin sensitivity in high-fat diet-fed Park2 knockout mice. Am J Physiol Endocrinol Metab 2016, 311, (1), E105-16.

Czaja, M. J.; Ding, W. X.; Donohue, T. M., Jr.; Friedman, S. L.; Kim, J. S.; Komatsu, M et al., Functions of autophagy in normal and diseased liver. Autophagy 2013, 9, (8), 1131-58.

Donati, A.; Taddei, M.; Cavallini, G.; Bergamini, E., Stimulation of macroautophagy can rescue older cells from 8-OHdG mtDNA accumulation: a safe and easy way to meet goals in the SENS agenda. Rejuvenation Res 2006, 9, (3), 408-12.

Dumas, K.; Ayachi, C.; Gilleron, J.; Lacas-Gervais, S.; Pastor, F.; Favier, F. B.; Peraldi, P.; Vaillant, N.; Yvan-Charvet, L.; Bonnafous, S.; Patouraux, S.; Anty, R.; Tran, A.; Gual, P.; Cormont, M.; Tanti, J. F.; Giorgetti-Peraldi, S., REDD1 deficiency protects against nonalcoholic hepatic steatosis induced by high-fat diet. FASEB J 2020, 34, (4), 5046-5060.

Edmunds, L. R.; Huckestein, B. R.; Kahn, M.; Zhang, D.; Chu, Y.; Zhang, Y.; Wendell, S. G.; Shulman, G. I.; Jurczak, M. J., Hepatic insulin sensitivity is improved in high-fat diet-fed Park2 knockout mice in association with increased hepatic AMPK activation and reduced steatosis. Physiol Rep 2019, 7, (21), e14281.

Eid N, Ito Y, Otsuki Y. Triggering of Parkin Mitochondrial Translocation in Mitophagy: Implications for Liver Diseases. Front Pharmacol. 2016a, 29;7:100.

Eid, N.; Ito, Y.; Horibe, A.; Otsuki, Y., Ethanol-induced mitophagy in liver is associated with activation of the PINK1-Parkin pathway triggered by oxidative DNA damage. Histol Histopathol 2016b, 31, (10), 1143-59.

Eid, N.; Ito, Y.; Maemura, K.; Otsuki, Y., Elevated autophagic sequestration of mitochondria and lipid droplets in steatotic hepatocytes of chronic ethanol-treated rats: an immunohistochemical and electron microscopic study. J Mol Histol 2013, 44, (3), 311-26.

Eid, N.; Ito, Y.; Otsuki, Y., Mitophagy in steatotic hepatocytes of ethanol-treated wild-type and Parkin knockout mice. Am J Physiol Gastrointest Liver Physiol 2015, 309, (6), G513-4.

Ezaki, J.; Matsumoto, N.; Takeda-Ezaki, M.; Komatsu, M.; Takahashi, K.; Hiraoka, Y et al. Liver autophagy contributes to the maintenance of blood glucose and amino acid levels. Autophagy 2011, 7, (7), 727-36.

Fukuo, Y.; Yamashina, S.; Sonoue, H.; Arakawa, A.; Nakadera, E.; Aoyama, T et al. Abnormality of autophagic function and cathepsin expression in the liver from patients with non-alcoholic fatty liver disease. Hepatol Res 2014, 44, (9), 1026-36.

Glick, D.; Zhang, W.; Beaton, M.; Marsboom, G.; Gruber, M.; Simon, M. C et al. BNip3 regulates mitochondrial function and lipid metabolism in the liver. Mol Cell Biol 2012, 32, (13), 2570-84.

Gonzalez-Rodriguez, A.; Mayoral, R.; Agra, N.; Valdecantos, M. P.; Pardo, V.; Miquilena-Colina, M. E et al. Impaired autophagic flux is associated with increased endoplasmic reticulum stress during the development of NAFLD. Cell Death Dis 2014, 5, e1179.

Gordon, E. R., Mitochondrial functions in an ethanol-induced fatty liver. J Biol Chem 1973, 248, (23), 8271-80.

Hara, Y.; Yanatori, I.; Ikeda, M.; Kiyokage, E.; Nishina, S.; Tomiyama, Y.; Toida, K.; Kishi, F.; Kato, N.; Imamura, M.; Chayama, K.; Hino, K., Hepatitis C virus core protein suppresses mitophagy by interacting with parkin in the context of mitochondrial depolarization. Am J Pathol 2014, 184, (11), 3026-39.

Hong, J. M.; Lee, S. M., Heme oxygenase-1 protects liver against ischemia/reperfusion injury via phosphoglycerate mutase family member 5-mediated mitochondrial quality control. Life Sci 2018, 200, 94-104.

Huang, Q.; Zhan, L.; Cao, H.; Li, J.; Lyu, Y.; Guo, X.; Zhang, J.; Ji, L.; Ren, T.; An, J.; Liu, B.; Nie, Y.; Xing, J., Increased mitochondrial fission promotes autophagy and hepatocellular carcinoma cell survival through the ROS-modulated coordinated regulation of the NFKB and TP53 pathways. Autophagy 2016, 12, (6), 999-1014.

Hur, J. H.; Park, S. Y.; Dall'Armi, C.; Lee, J. S.; Di Paolo, G.; Lee, H. Y.et al. Phospholipase D1 deficiency in mice causes nonalcoholic fatty liver disease via an autophagy defect. Sci Rep 2016, 6, 39170.

Inami, Y.; Yamashina, S.; Izumi, K.; Ueno, T.; Tanida, I.; Ikejima, K.; Watanabe, S., Hepatic steatosis inhibits autophagic proteolysis via impairment of autophagosomal acidification and cathepsin expression. Biochem Biophys Res Commun 2011, 412, (4), 618-25.

Jassey, A.; Liu, C. H.; Changou, C. A.; Richardson, C. D.; Hsu, H. Y.; Lin, L. T., Hepatitis C Virus Non-Structural Protein 5A (NS5A) Disrupts Mitochondrial Dynamics and Induces Mitophagy. Cells 2019, 8, (4).

Ji, E.; Kim, C.; Kang, H.; Ahn, S.; Jung, M.; Hong, Y. et al. RNA Binding Protein HuR Promotes Autophagosome Formation by Regulating Expression of Autophagy-Related Proteins 5, 12, and 16 in Human Hepatocellular Carcinoma Cells. Mol Cell Biol 2019, 39, (6).

Kang, J. W.; Hong, J. M.; Lee, S. M., Melatonin enhances mitophagy and mitochondrial biogenesis in rats with carbon tetrachloride-induced liver fibrosis. J Pineal Res 2016, 60, (4), 383-93.
Ke, P. Y., Mitophagy in the Pathogenesis of Liver Diseases. Cells 2020, 9, (4).
Kharbanda, K. K.; McVicker, D. L.; Zetterman, R. K.; Donohue, T. M., Jr., Ethanol consumption alters trafficking of lysosomal enzymes and affects the processing of procathepsin L in rat liver. Biochim Biophys Acta 1996, 1291, (1), 45-52.
Kheloufi, M.; Boulanger, C. M.; Durand, F.; Rautou, P. E., Liver autophagy in anorexia nervosa and acute liver injury. Biomed Res Int 2014, 2014, 701064.
Kim, I.; Lemasters, J. J., Mitochondrial degradation by autophagy (mitophagy) in GFP-LC3 transgenic hepatocytes during nutrient deprivation. Am J Physiol Cell Physiol 2011, 300, (2), C308-17.
Kim, J. S.; Nitta, T.; Mohuczy, D.; O'Malley, K. A.; Moldawer, L. L.; Dunn, W. A., Jr.; Behrns, K. E., Impaired autophagy: A mechanism of mitochondrial dysfunction in anoxic rat hepatocytes. Hepatology 2008, 47, (5), 1725-36.
Kim, S. J.; Khan, M.; Quan, J.; Till, A.; Subramani, S.; Siddiqui, A., Hepatitis B virus disrupts mitochondrial dynamics: induces fission and mitophagy to attenuate apoptosis. PLoS Pathog 2013a, 9, (12), e1003722.
Kim, S. J.; Syed, G. H.; Khan, M.; Chiu, W. W.; Sohail, M. A.; Gish, R. G.; Siddiqui, A., Hepatitis C virus triggers mitochondrial fission and attenuates apoptosis to promote viral persistence. Proc Natl Acad Sci U S A 2014, 111, (17), 6413-8.
Kim, S. J.; Syed, G. H.; Siddiqui, A., Hepatitis C virus induces the mitochondrial translocation of Parkin and subsequent mitophagy. PLoS Pathog 2013b, 9, (3), e1003285.
Koga, H.; Kaushik, S.; Cuervo, A. M., Altered lipid content inhibits autophagic vesicular fusion. FASEB J 2010, 24, (8), 3052-65.
Komatsu, M.; Waguri, S.; Koike, M.; Sou, Y. S.; Ueno, T.; Hara et al. Homeostatic levels of p62 control cytoplasmic inclusion body formation in autophagy-deficient mice. Cell 2007, 131, (6), 1149-63.
Komatsu, M.; Waguri, S.; Ueno, T.; Iwata, J.; Murata, S.; Tanida, I et al. Impairment of starvation-induced and constitutive autophagy in Atg7-deficient mice. J Cell Biol 2005, 169, (3), 425-34.
Lee, K.; Haddad, A.; Osme, A.; Kim, C.; Borzou, A.; Ilchenko, S.; Allende, D.; Dasarathy, S.; McCullough, A.; Sadygov, R. G.; Kasumov, T., Hepatic Mitochondrial Defects in a Nonalcoholic Fatty Liver Disease Mouse Model Are Associated with Increased Degradation of Oxidative Phosphorylation Subunits. Mol Cell Proteomics 2018, 17, (12), 2371-2386.
Lei, H. Y.; Chang, C. P., Induction of autophagy by concanavalin A and its application in anti-tumor therapy. Autophagy 2007, 3, (4), 402-4.
Li, S.; Dou, X.; Ning, H.; Song, Q.; Wei, W.; Zhang, X.; et al. Sirtuin 3 acts as a negative regulator of autophagy dictating hepatocyte susceptibility to lipotoxicity. Hepatology 2017, 66, (3), 936-952.
Li, W.; Li, Y.; Siraj, S.; Jin, H.; Fan, Y.; Yang, X.; Huang, X.; Wang, X.; Wang, J.; Liu, L.; Du, L.; Chen, Q., FUN14 Domain-Containing 1-Mediated Mitophagy Suppresses Hepatocarcinogenesis by Inhibition of Inflammasome Activation in Mice. Hepatology 2019, 69, (2), 604-621.
Li, Y.; Ruan, D. Y.; Jia, C. C.; Zheng, J.; Wang, G. Y.; Zhao, H. et al. Aging aggravates hepatic ischemia-reperfusion injury in mice by impairing mitophagy with the involvement of the EIF2alpha-parkin pathway. Aging (Albany NY) 2018, 10, (8), 1902-1920.
Liu, H. Y.; Han, J.; Cao, S. Y.; Hong, T.; Zhuo, D.; Shi, J.; Liu, Z.; Cao, W., Hepatic autophagy is suppressed in the presence of insulin resistance and hyperinsulinemia: inhibition of FoxO1-dependent expression of key autophagy genes by insulin. J Biol Chem 2009, 284, (45), 31484-92.
Liu, K.; Lee, J.; Kim, J. Y.; Wang, L.; Tian, Y.; Chan, S. T.; Cho, C.; Machida, K.; Chen, D.; Ou, J. J., Mitophagy Controls the Activities of Tumor Suppressor p53 to Regulate Hepatic Cancer Stem Cells. Mol Cell 2017, 68, (2), 281-292 e5.
Liu, K.; Shi, Y.; Guo, X. H.; Ouyang, Y. B.; Wang, S. S.; Liu, D. J.; Wang, A. N.; Li, N.; Chen, D. X., Phosphorylated AKT inhibits the apoptosis induced by DRAM-mediated mitophagy in hepatocellular carcinoma by preventing the translocation of DRAM to mitochondria. Cell Death Dis 2014, 5, e1078.

Menk, M.; Graw, J. A.; Poyraz, D.; Mobius, N.; Spies, C. D.; von Haefen, C., Chronic Alcohol Consumption Inhibits Autophagy and Promotes Apoptosis in the Liver. Int J Med Sci 2018, 15, (7), 682-688.

Ning, X. J.; Yan, X.; Wang, Y. F.; Wang, R.; Fan, X. L.; Zhong, Z. B.; Ye, Q. F., Parkin deficiency elevates hepatic ischemia/reperfusion injury accompanying decreased mitochondrial autophagy, increased apoptosis, impaired DNA damage repair and altered cell cycle distribution. Mol Med Rep 2018, 18, (6), 5663-5668.

Niture, S.; Lin, M.; Rios-Colon, L.; Qi, Q.; Moore, J. T.; Kumar, D., Emerging Roles of Impaired Autophagy in Fatty Liver Disease and Hepatocellular Carcinoma. Int J Hepatol 2021, 2021, 6675762.

Pang, L.; Liu, K.; Liu, D.; Lv, F.; Zang, Y.; Xie, F.; Yin, J.; Shi, Y.; Wang, Y.; Chen, D., Differential effects of reticulophagy and mitophagy on nonalcoholic fatty liver disease. Cell Death Dis 2018, 9, (2), 90.

Park, H. W.; Park, H.; Semple, I. A.; Jang, I.; Ro, S. H.; Kim, M et al., Pharmacological correction of obesity-induced autophagy arrest using calcium channel blockers. Nat Commun 2014, 5, 4834.

Perlmutter, D. H., Liver injury in alpha1-antitrypsin deficiency: an aggregated protein induces mitochondrial injury. J Clin Invest 2002, 110, (11), 1579-83.

Pi, H.; Xu, S.; Zhang, L.; Guo, P.; Li, Y.; Xie, J.; Tian, L.; He, M.; Lu, Y.; Li, M.; Zhang, Y.; Zhong, M.; Xiang, Y.; Deng, L.; Zhou, Z.; Yu, Z., Dynamin 1-like-dependent mitochondrial fission initiates overactive mitophagy in the hepatotoxicity of cadmium. Autophagy 2013, 9, (11), 1780-800.

Prieto-Dominguez, N.; Ordonez, R.; Fernandez, A.; Mendez-Blanco, C.; Baulies, A.; Garcia-Ruiz, C.; Fernandez-Checa, J. C.; Mauriz, J. L.; Gonzalez-Gallego, J., Melatonin-induced increase in sensitivity of human hepatocellular carcinoma cells to sorafenib is associated with reactive oxygen species production and mitophagy. J Pineal Res 2016, 61, (3), 396-407.

Qian, H.; Yang, Y., Alterations of cellular organelles in human liver-derived hepatoma G2 cells induced by adriamycin. Anticancer Drugs 2009, 20, (9), 779-86.

Qian, H.; Yang, Y.; Wang, X., Curcumin enhanced adriamycin-induced human liver-derived Hepatoma G2 cell death through activation of mitochondria-mediated apoptosis and autophagy. Eur J Pharm Sci 2011, 43, (3), 125-31.

Qiu, Y. N.; Wang, G. H.; Zhou, F.; Hao, J. J.; Tian, L.; Guan, L. F.; Geng, X. K.; Ding, Y. C.; Wu, H. W.; Zhang, K. Z., PM2.5 induces liver fibrosis via triggering ROS-mediated mitophagy. Ecotoxicol Environ Saf 2019, 167, 178-187.

Rasineni, K.; Donohue, T. M., Jr.; Thomes, P. G.; Yang, L.; Tuma, D. J.; McNiven, M. A.; Casey, C. A., Ethanol-induced steatosis involves impairment of lipophagy, associated with reduced Dynamin2 activity. Hepatol Commun 2017, 1, (6), 501-512.

Rodriguez-Enriquez, S.; Kai, Y.; Maldonado, E.; Currin, R. T.; Lemasters, J. J., Roles of mitophagy and the mitochondrial permeability transition in remodeling of cultured rat hepatocytes. Autophagy 2009, 5, (8), 1099-106.

Schulze, R. J.; Rasineni, K.; Weller, S. G.; Schott, M. B.; Schroeder, B.; Casey, C. A.; McNiven, M. A., Ethanol exposure inhibits hepatocyte lipophagy by inactivating the small guanosine triphosphatase Rab7. Hepatol Commun 2017, 1, (2), 140-152.

Shan, S.; Shen, Z.; Zhang, C.; Kou, R.; Xie, K.; Song, F., Mitophagy protects against acetaminophen-induced acute liver injury in mice through inhibiting NLRP3 inflammasome activation. Biochem Pharmacol 2019, 169, 113643.

Singh, B. K.; Sinha, R. A.; Tripathi, M.; Mendoza, A.; Ohba, K.; Sy, J. A. C.; Xie, S. Y.; Zhou, J.; Ho, J. P.; Chang, C. Y.; Wu, Y.; Giguere, V.; Bay, B. H.; Vanacker, J. M.; Ghosh, S.; Gauthier, K.; Hollenberg, A. N.; McDonnell, D. P.; Yen, P. M., Thyroid hormone receptor and ERRalpha coordinately regulate mitochondrial fission, mitophagy, biogenesis, and function. Sci Signal 2018, 11, (536).

Sinha, R. A.; Yen, P. M., Thyroid hormone-mediated autophagy and mitochondrial turnover in NAFLD. Cell Biosci 2016, 6, 46.

Sun, X. L.; Zhang, Y. L.; Xi, S. M.; Ma, L. J.; Li, S. P., MiR-330-3p suppresses phosphoglycerate mutase family member 5 -inducted mitophagy to alleviate hepatic ischemia-reperfusion injury. J Cell Biochem 2019, 120, (3), 4255-4267.

Takamura, A.; Komatsu, M.; Hara, T.; Sakamoto, A.; Kishi, C.; Waguri, S et al. Autophagy-deficient mice develop multiple liver tumors. Genes Dev 2011, 25, (8), 795-800.

Tanaka, S.; Hikita, H.; Tatsumi, T.; Sakamori, R.; Nozaki, Y.; Sakane, S. et al. Rubicon inhibits autophagy and accelerates hepatocyte apoptosis and lipid accumulation in nonalcoholic fatty liver disease in mice. Hepatology 2016, 64, (6), 1994-2014.

Teckman, J. H.; An, J. K.; Blomenkamp, K.; Schmidt, B.; Perlmutter, D., Mitochondrial autophagy and injury in the liver in alpha 1-antitrypsin deficiency. Am J Physiol Gastrointest Liver Physiol 2004, 286, (5), G851-62.

Tian, Y.; Kuo, C. F.; Sir, D.; Wang, L.; Govindarajan, S.; Petrovic, L. M.; Ou, J. H., Autophagy inhibits oxidative stress and tumor suppressors to exert its dual effect on hepatocarcinogenesis. Cell Death Differ 2015, 22, (6), 1025-34.

Tian, Z.; Chen, Y.; Yao, N.; Hu, C.; Wu, Y.; Guo, D.; Liu, J.; Yang, Y.; Chen, T.; Zhao, Y.; He, Y., Role of mitophagy regulation by ROS in hepatic stellate cells during acute liver failure. Am J Physiol Gastrointest Liver Physiol 2018, 315, (3), G374-G384.

Wang, L.; Liu, X.; Nie, J.; Zhang, J.; Kimball, S. R.; Zhang, H.; Zhang, W. J.; Jefferson, L. S.; Cheng, Z.; Ji, Q.; Shi, Y., ALCAT1 controls mitochondrial etiology of fatty liver diseases, linking defective mitophagy to steatosis. Hepatology 2015, 61, (2), 486-96.

Williams, J. A.; Ding, W. X., Mitophagy, mitochondrial spheroids, and mitochondrial-derived vesicles in alcohol-induced liver injury. Am J Physiol Gastrointest Liver Physiol 2015a, 309, (6), G515.

Williams, J. A.; Ni, H. M.; Ding, Y.; Ding, W. X., Parkin regulates mitophagy and mitochondrial function to protect against alcohol-induced liver injury and steatosis in mice. Am J Physiol Gastrointest

Willy, J. A.; Young, S. K.; Mosley, A. L.; Gawrieh, S.; Stevens, J. L.; Masuoka, H. C.; Wek, R. C., Function of inhibitor of Bruton's tyrosine kinase isoform alpha (IBTKalpha) in nonalcoholic steatohepatitis links autophagy and the unfolded protein response. J Biol Chem 2017, 292, (34), 14050-14065.

Wu, H.; Chen, G.; Wang, J.; Deng, M.; Yuan, F.; Gong, J., TIM-4 interference in Kupffer cells against CCL4-induced liver fibrosis by mediating Akt1/Mitophagy signalling pathway. Cell Prolif 2020, 53, (1), e12731.

Wu, H.; Wang, Y.; Li, W.; Chen, H.; Du, L.; Liu, D.; Wang, X.; Xu, T.; Liu, L.; Chen, Q., Deficiency of mitophagy receptor FUNDC1 impairs mitochondrial quality and aggravates dietary-induced obesity and metabolic syndrome. Autophagy 2019, 15, (11), 1882-1898.

Yamada, T.; Murata, D.; Adachi, Y.; Itoh, K.; Kameoka, S.; Igarashi, A.; Kato, T.; Araki, Y.; Huganir, R. L.; Dawson, T. M.; Yanagawa, T.; Okamoto, K.; Iijima, M.; Sesaki, H., Mitochondrial Stasis Reveals p62-Mediated Ubiquitination in Parkin-Independent Mitophagy and Mitigates Nonalcoholic Fatty Liver Disease. Cell Metab 2018, 28, (4), 588-604 e5.

You, M.; Matsumoto, M.; Pacold, C. M.; Cho, W. K.; Crabb, D. W., The role of AMP-activated protein kinase in the action of ethanol in the liver. Gastroenterology 2004, 127, (6), 1798-808.

Yue, Z.; Jin, S.; Yang, C.; Levine, A. J.; Heintz, N., Beclin 1, an autophagy gene essential for early embryonic development, is a haploinsufficient tumor suppressor. Proc Natl Acad Sci U S A 2003, 100, (25), 15077-82.

Zhang, N. P.; Liu, X. J.; Xie, L.; Shen, X. Z.; Wu, J., Impaired mitophagy triggers NLRP3 inflammasome activation during the progression from nonalcoholic fatty liver to nonalcoholic steatohepatitis. Lab Invest 2019, 99, (6), 749-763.

Zhou, H.; Zhu, P.; Wang, J.; Toan, S.; Ren, J., DNA-PKcs promotes alcohol-related liver disease by activating Drp1-related mitochondrial fission and repressing FUNDC1-required mitophagy. Signal Transduct Target Ther 2019, 4, 56.

Zubiete-Franco, I.; Garcia-Rodriguez, J. L.; Martinez-Una, M.; Martinez-Lopez, N.; Woodhoo, A.; Juan, et al., Methionine and S-adenosylmethionine levels are critical regulators of PP2A activity modulating lipophagy during steatosis. J Hepatol 2016, 64, (2), 409-418.

Supplementary Figure 1

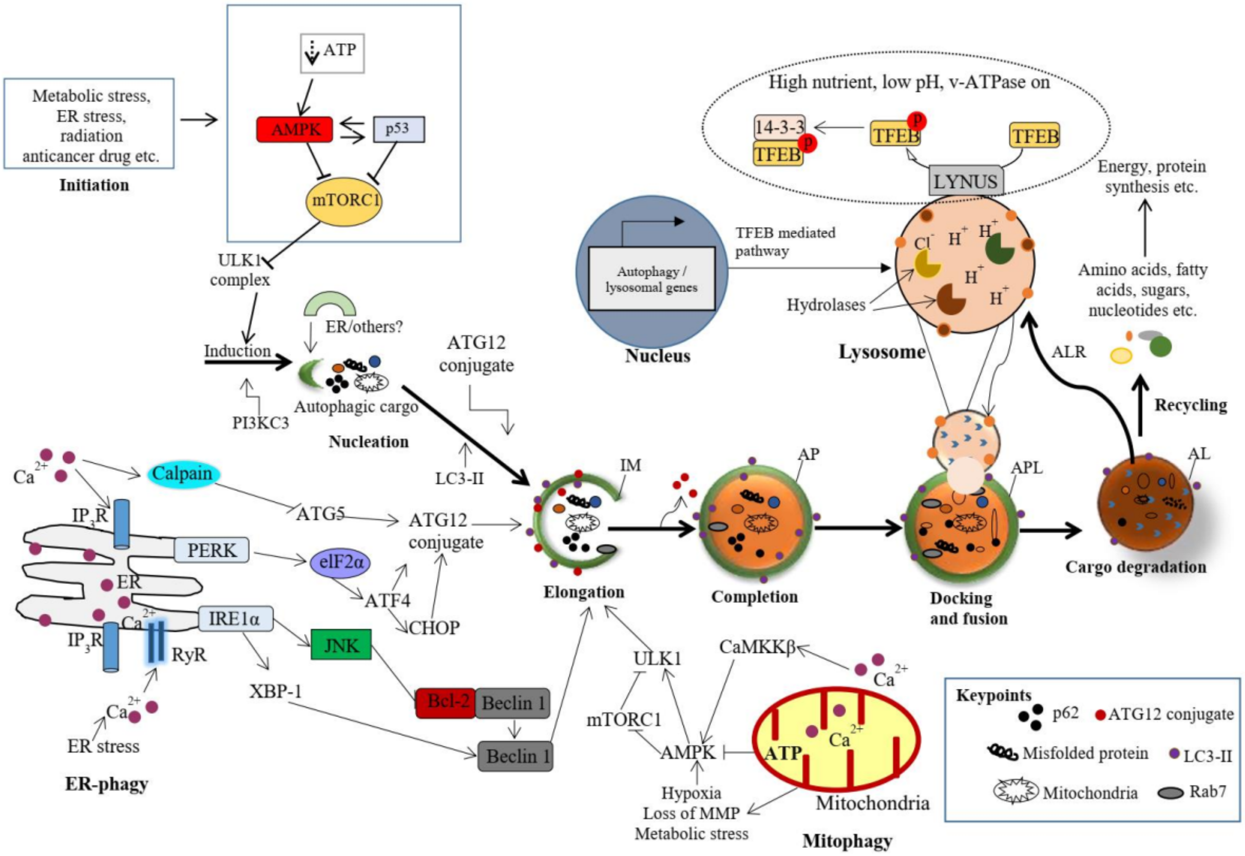

Figure S1 legend. **Molecular mechanisms of various stages of autophagy**. Autophagy is activated in response to various cellular stresses and is triggered by a decrease in rapamycin complex 1 (mTORC1) activity due to the activation of AMP-activated protein kinase (AMPK) or p53 signaling. mTORC1 suppresses the activity of Unc-51-like autophagy activating kinase 1 (ULK1) complex. Therefore, inhibition of mTORC1 causes the initialization of the ULK1-mediated formation of the isolation (autophagosomal) membrane (IM) in association with the class III phosphatidylinositide 3-kinase (PI3K) complex (PI3KC3). The IM expands into an autophagosome (AP) with a double-layer membrane, which can engulf any cellular component, including proteins, damaged organelles, and lipid droplets. The AP merges with the lysosome (via LAMP-1, 2), forming autophagolysosome (APL) or autolysosome (AL), and resulting in the degradation of the cargo by cathepsins and the autophagic lysosome reformation (ALR). The nucleation, elongation and maturation of the IM are dependent on two ubiquitin-like conjugation systems (ATG12 and ATG8), which involve multiple autophagy proteins, including Beclin1, ATG5, ATG16 and MT-associated protein 1 light chain 3 (LC3). The AL provides an acidic milieu for hydrolytic

enzymes to digest the engulfed components. Nuclear localization of transcription factor EB (TFEB) is critical to the formation of lysosomes and to the enhanced expression of autophagy proteins. Importantly, autophagy could be selective of mitochondria (mitophagy) or ER (ER-phagy). (Al-Bari et al 2021, reprinted with permission from IJMS).
